# Engineering of Integral Membrane Metalloenzyme UndB and Designing of a Cell-Free Biocatalytic Platform Enabled Efficient 1‑Alkene Production

**DOI:** 10.1021/acscentsci.5c01099

**Published:** 2025-10-14

**Authors:** Tabish Iqbal, Subhashini Murugan, Jayaprakash Karupusamy, Abhishek Sirohiwal, Debasis Das

**Affiliations:** Department of Inorganic and Physical Chemistry, 29120 Indian Institute of Science, Bangalore, Karnataka-560012, India

## Abstract

The bioproduction of 1-alkenes is of significant global interest due to their potential as green commodity chemicals and next-generation ‘drop-in’ biofuels. Here, we report an engineering strategy to enhance the catalytic activity and substrate specificity of the membrane-bound metalloenzyme UndB, significantly improving its utility in biocatalytic 1-alkene production. We developed a highly efficient UndB-based cell-free biocatalytic platform for high-yield medium-chain 1-alkene production. This system achieved a 262-fold improvement in UndB activity toward 1-undecene production, with a total turnover of 3412. Through structural analysis of the UndB family of proteins, we engineered UndB by domain-swapping, enhancing its selectivity toward naturally abundant long-chain fatty acids, enabling efficient long-chain 1-alkene production. Our large-scale simulations unveiled a crucial ion-pair network that orchestrates substrate–protein interactions, providing a framework for substrate stabilization. We identified a highly dynamic and functionally pivotal Arg121 residue that governs substrate uptake and stabilization, providing mechanistic insights into UndB’s substrate recognition. Furthermore, simulations revealed that precise modulation of the substrate-binding pocket volume serves as the key determinant of substrate specificity across UndB variants, offering insights into the evolutionary adaptability of the UndB family. Our system achieved 98% 1-alkene yield using only 0.04 mol % catalyst loading under mild conditions, presenting a promising bioproduction strategy.

## Graphical abstract

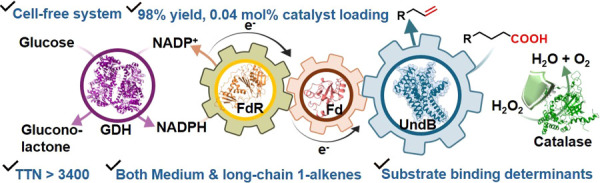

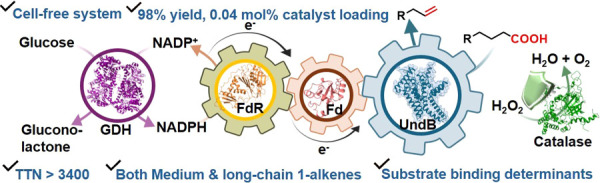

## Introduction

The development of alternatives to traditional petroleum-based chemicals and fuels is crucial for a greener future.^1–4^ Conventional methods for producing commodity chemicals rely heavily on fossil fuels, involve harsh reaction conditions, use expensive metal catalysts, and generate undesired byproducts^5–10^ (Figure 1). These practices pose significant environmental and efficiency challenges. Biomass, particularly abundant natural resources like fatty acids, presents a promising renewable feedstock for generating fuels and green chemicals, including alkanes and alkenes.^11–13^ However, the existing physiochemical methods for converting fatty acids into hydrocarbons face substantial challenges and limitations (Figure 1),^9^ highlighting the need for sustainable and environmentally friendly alternatives.

**Figure 1:**
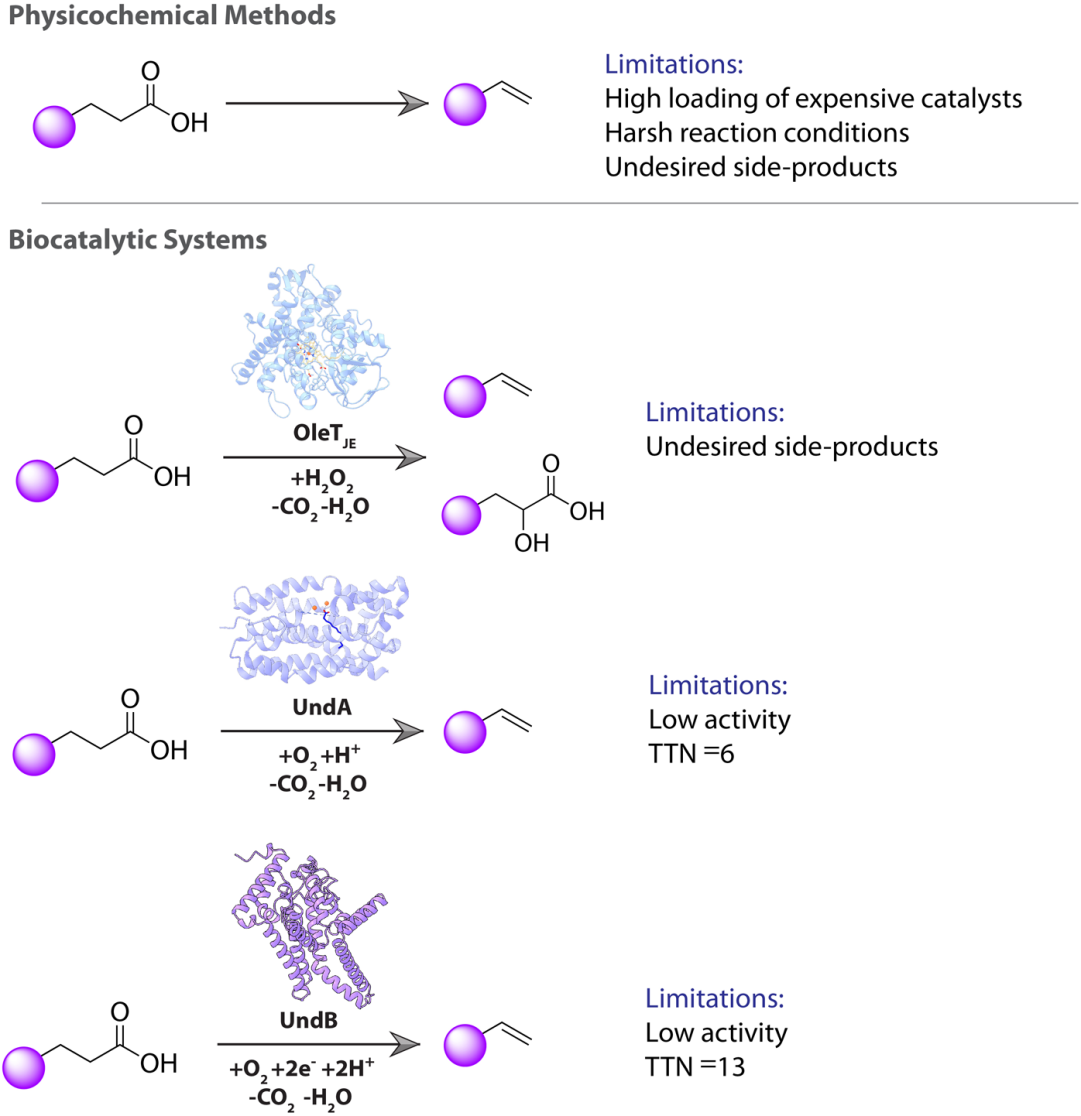
Overview of the physiochemical methods and biocatalytic systems for 1-alkene production. Physicochemical methods for 1-alkene production require expensive metal catalysts and harsh reaction conditions, leading to undesired side-products. The heme-containing OleT enzyme-based biocatalytic system requires H_2_O_2_ to convert fatty acids into 1-alkenes. This system produces hydroxy fatty acids as undesired side-products. The diiron-containing enzyme UndA requires molecular oxygen as a co-substrate and can perform a single catalytic turnover without the need for external redox partners or electron donors. UndA shows very poor activity, with a maximum TTN of 6. The transmembrane enzyme UndB requires O_2_ and a reductive electron system to provide two reducing equivalents to catalyze 1-alkene production. The purified UndB suffers from poor activity, with a TTN of 13.

Enzyme-based systems offer a promising biocatalytic approach to overcoming these challenges, as enzymes can perform challenging chemical reactions under mild conditions with high selectivity and specificity.^14^ One such enzyme is UndB, capable of converting fatty acids into 1-alkenes in a single-step reaction under mild conditions^15,16^ (Figure 1). UndB is an integral membrane metalloenzyme that belongs to the superfamily of fatty acid desaturases (FADS superfamily).^16,17^ The members of the FADS superfamily are characterized by their localization in cellular membranes and a nonheme diiron catalytic center at their active site. These enzymes are proposed to require oxygen and reductive co-substrates to catalyze chemically challenging reactions such as desaturation, hydroxylation, decarboxylation, etc.^17–19^ Our previous research also confirms that UndB also needs oxygen and a reductive electron transfer system to perform the unique decarboxylation of fatty acids to produce 1-alkenes^16^ (Figure 1). This UndB-based oxidative and redox-dependent biocatalytic route presents a potentially sustainable and selective alternative to the traditional chemical methods of alkene production.^3,13^ Recently, our group and others have also demonstrated that microbial strains expressing UndB can efficiently produce medium-chain 1-alkenes from corresponding fatty acids.^20,21^ However, the low activity of isolated UndB with a total turnover number (TTN) of 13 ^16^ remains a bottleneck, restricting its large-scale applications. In our previous work, we improved the total activity of purified UndB by fusing the *katE* gene, which encodes *E. coli* catalase, to the N-terminus of UndB.^20^ The resulting chimeric protein KatE-UndB led to an enhancement of TTN by 19-fold (∼270 TTN).^20^

In this study, by synergistic coupling of UndB with two co-substrate recycling systems, we developed a robust cell-free biocatalytic (CFB) platform that maximizes the activity of this challenging membrane enzyme, achieving an unprecedented TTN of 3412 ± 254 for the bioproduction of 1-undecene, setting a new benchmark for medium-chain 1-alkene production using UndB. However, the overall productivity of long-chain 1-alkenes remains modest in comparison. To address this, we explored the structural diversity of UndB homologues and identified a key structural determinant for the enzyme’s substrate specificity. Subsequently, we performed strategic engineering of this structural motif, resulting in an engineered UndB exhibiting greater activity with long-chain fatty acids, which are naturally more abundant fatty acids, allowing UndB to produce biotechnologically important long-chain 1-alkenes efficiently. Furthermore, through large-scale molecular dynamics (MD) simulations, we elucidated the molecular basis for the substrate specificity among UndB homologues.

## Results and Discussion

### Co-substrate Recycling Boosts the Activity of the UndB-Based Biocatalytic System

Our group has recently shed light on several biochemical properties and catalytic details of UndB.^16,17,20^ We have established that UndB is a nonheme diiron-containing integral membrane enzyme capable of converting a range of free C_*n*_ fatty acids into the corresponding C_*n*–1_ 1-alkenes.^16^ We have also demonstrated that UndB catalysis requires O_2_ and a reductive electron transfer system as co-substrates and identified the cyanobacterial *S. elongatus* ferredoxin/ferredoxin reductase/NADPH system as the most effective reductive co-substrates.^16^ Despite the significant potential of UndB in producing biofuels and green commodity chemicals, we observed that UndB undergoes rapid inhibition by hydrogen peroxide (H_2_O_2_),^20^ produced *in situ* in the UndB reaction by uncoupled electron transfer from NADPH to molecular oxygen, resulting in a poor TTN of 13.^16^ Subsequently, our measurement of NADPH consumption in the UndB reaction revealed that nearly four equivalents of NADPH were consumed to produce only one equivalent of 1-alkene, leading to the wastage of three equivalents of this costly reducing agent.^20^ This inefficiency in 1-alkene production relative to the expensive reducing equivalents consumed significantly limits the applicability of UndB for large-scale 1-alkene production.

To address these issues, we hypothesized that using a minimal amount of the reducing equivalents and recycling them through a regeneration system could mitigate the need for large quantities of NADPH in the reaction and minimize the *in situ* production of H_2_O_2_, thereby enhancing the overall activity of UndB for alkene production. To test this, we employed a NADPH-regeneration system consisting of glucose dehydrogenase (GDH) and glucose to regenerate NADPH during the UndB reaction. However, the preliminary activity studies of *Pme*-UndB (UndB from *Pseudomonas mendocina* strain *ymp* (gene locus tag *Pmen_4370*)) with lauric acid revealed only a modest improvement in UndB activity (TTN = 23) with the regeneration system and 100 μM NADPH, compared to the standard UndB reaction (TTN = 13) with 1 mM NADPH without the regeneration system (Figure S1). These results support our hypothesis of enhancing the efficacy of UndB with the NADPH-regeneration system, albeit marginally. To further improve enzyme activity, we employed our previously engineered chimeric enzyme, KatE-UndB, which consists of *E. coli* catalase (KatE) conjugated at the N-terminus of *Pme*-UndB.^20^ This fusion construct was designed to mitigate H_2_O_2_-mediated inhibition of UndB by rapidly converting H_2_O_2_ into O_2_, the co-substrate for UndB. Notably, in the present work, the chimeric enzyme, in the presence of the NADPH-regeneration system, showed a substantial increase in activity (TTN = 915) during the first 60 min of the reaction (Figure S1). We concluded that the combination of NADPH recycling by the regeneration system and inhibitor metabolism by catalase significantly enhanced the overall activity of *Pme*-UndB (Figure 2A).

**Figure 2:**
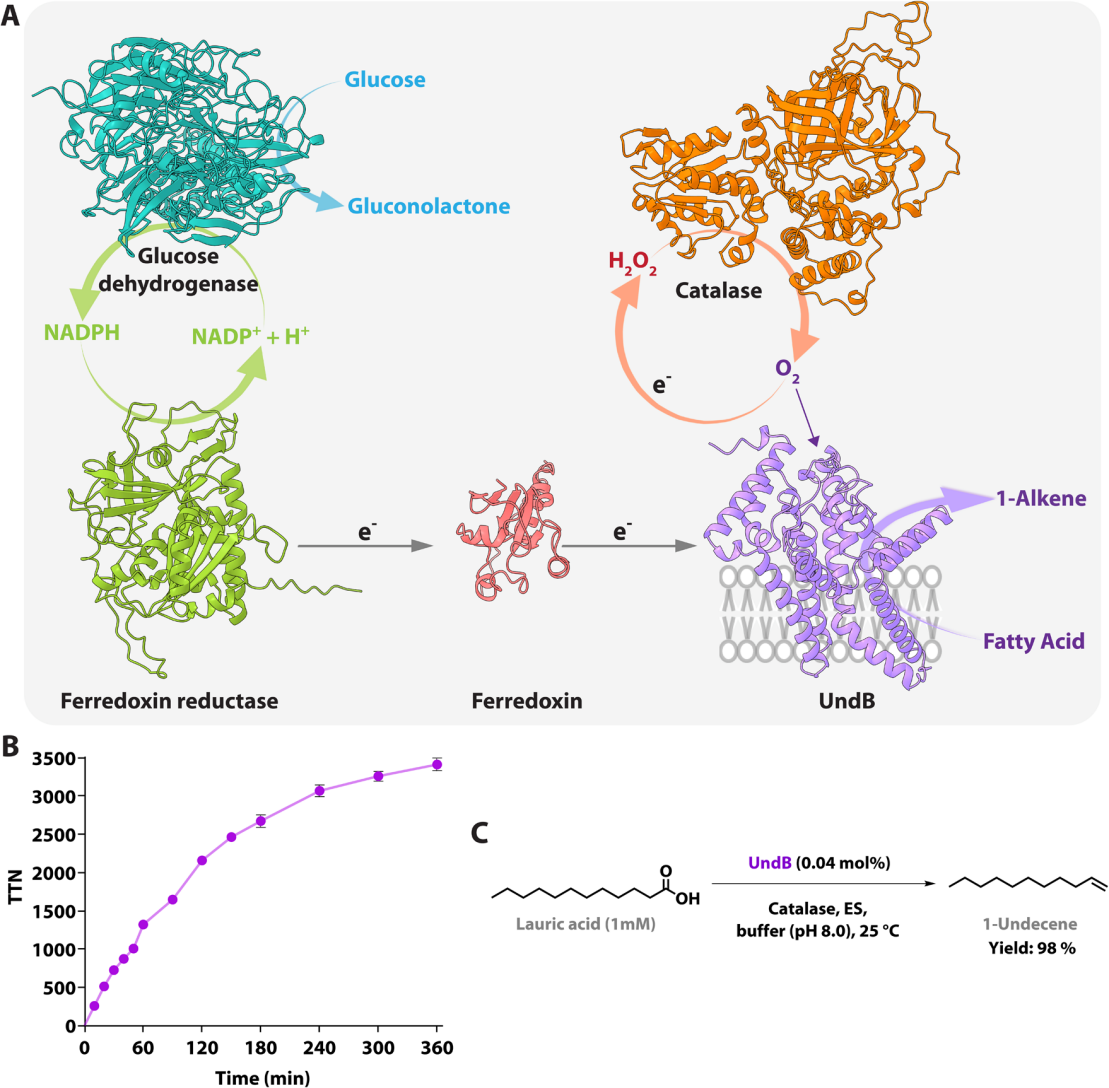
UndB-based CFB platform for efficient 1-alkene production. (A) Graphical representation of the UndB-based CFB system developed in this study. This system comprises UndB, catalase, and a reductive electron transfer system (ES) made of ferredoxin, ferredoxin reductase, NADPH, glucose dehydrogenase, and d-glucose. (B) Time-course of the CFB system, demonstrating its nearly linear activity in 1-undecene production for 360 min compared to the initially reported 1 min activity of UndB^16^ and 40 min activity of KatE-UndB (or UndB in the presence of external catalase) without the regeneration system.^20^ Error bars represent the standard deviation (SD) of triplicate data (*n* = 3). (C) Schematic of the quantitative conversion of lauric acid to 1-undecene using the CFB system.

### Development of a Cell-Free Biocatalytic Platform for Efficient 1-Undecene Production

To circumvent the challenges associated with purifying the transmembrane enzyme UndB (or KatE-UndB)—a process that is labor-intensive, requires expensive detergents, and results in substantial protein loss^22^—we sought to develop a user-friendly cell-free biocatalytic (CFB) system for efficient 1-alkene production. In our studies, we utilized the cellular membrane envelope fractions (CEF) from *E. coli* expressing *Pme*-UndB. Notably, the CEF can be readily prepared by lysing the cells expressing UndB followed by centrifugation (see Experimental Section) with minimal protein loss. Such CFB systems offer several advantages over whole-cell biocatalysts, including precise control over reaction conditions, faster reaction rates due to the absence of cellular regulatory barriers, and easier optimization and direct incorporation of cofactor regeneration systems, thereby enhancing performance.

Optimization studies revealed that the reaction conditions for maximum 1-undecene production from lauric acid involve merely 0.1 μM *Pme*-UndB (in CEF) in the presence of 1 U/mL catalase (for mitigating peroxide-mediated inhibition), 15 μM ferredoxin, 2.5 μM ferredoxin reductase, and 10 U/mL GDH in the presence of 2 mM glucose (Figure S2). Additionally, the system achieved the maximum activity with only 200 μM NADPH (Figure S2), significantly reducing the reliance on high concentrations of this costly cofactor. A time course of the *Pme*-UndB reaction under these optimized conditions showed a steady increase in 1-undecene production substantially over 6 h, compared to only 40 min observed previously,^20^ reaching a final TTN of 3412 ± 254 (Figure 2B). This represents a remarkable 262-fold improvement of *Pme*-UndB activity compared to the initially reported values in the absence of the cofactor recycling systems.^16^ This CFB system also significantly enhanced the turnover frequency of UndB, improving from 8 min^–1^ ^20^ to 24 min^–1^ over the initial 30 min. We further evaluated the performance of this system in converting lauric acid (at 1 mM scale) into 1-undecene and found that the CFB system achieved an impressive 98% yield with an exceptionally low catalyst loading (0.04%) under mild reaction conditions (25 °C, pH 8.0) (Figure 2C). These results underscore the tremendous potential of the UndB-based CFB system for highly efficient and high-yield production of 1-undecene.

### Efficient Production of 1-Alkenes Using the Cell-Free Biocatalytic Platform

Next, we investigated the effectiveness of the CFB system for converting fatty acids of various chain lengths to the corresponding 1-alkenes (C5–C17). These alkenes, beyond their potential as next-generation biofuels, have diverse industrial usage:^23,24^ short-chain alkenes (C6–C10) are crucial building blocks for petrochemicals and plastics;^25^ medium-chain alkenes are employed for producing detergents and lubricants;^26,27^ and long-chain alkenes are essential components in biomembranes, cosmetics, and pharmaceuticals.^28,29^ We observed that our CFB system exhibits a significantly improved activity in producing a wide range of 1-alkenes differing in their chain lengths (C5–C17) compared to previous studies^16^ (Figure 3). Notably, it achieved remarkable activity in producing medium-chain 1-alkenes (C9–C13) with a TTN of 2313 ± 11 for 1-nonene, 3412 ± 254 for 1-undecene, and 1960 ± 40 for 1-tridecene. Additionally, we observed that *Pme*-UndB demonstrated better 1-alkene production for short-chain fatty acids (C6–C10) at 4 °C compared to 25 °C (Figure S3), likely due to the volatility of these short-chain alkenes, which aligns with previous findings.^30^ While the activity of UndB for producing long-chain 1-alkenes (C15–C17) also improved significantly, productivity remained modest, which we substantially enhanced further through targeted enzyme engineering, as described below. Importantly, in comparison to the previously developed biocatalytic system based on OleT—a soluble heme-containing fatty acid decarboxylase^30^ —our UndB-based system achieved a substantially superior TTN of 3412 ± 254, representing the highest productivity of 1-undecene production achieved to date. Overall, our results highlight the potential of this biocatalytic platform to efficiently convert naturally abundant and inexpensive fatty acids into a wide array of highly valuable 1-alkenes.

**Figure 3:**
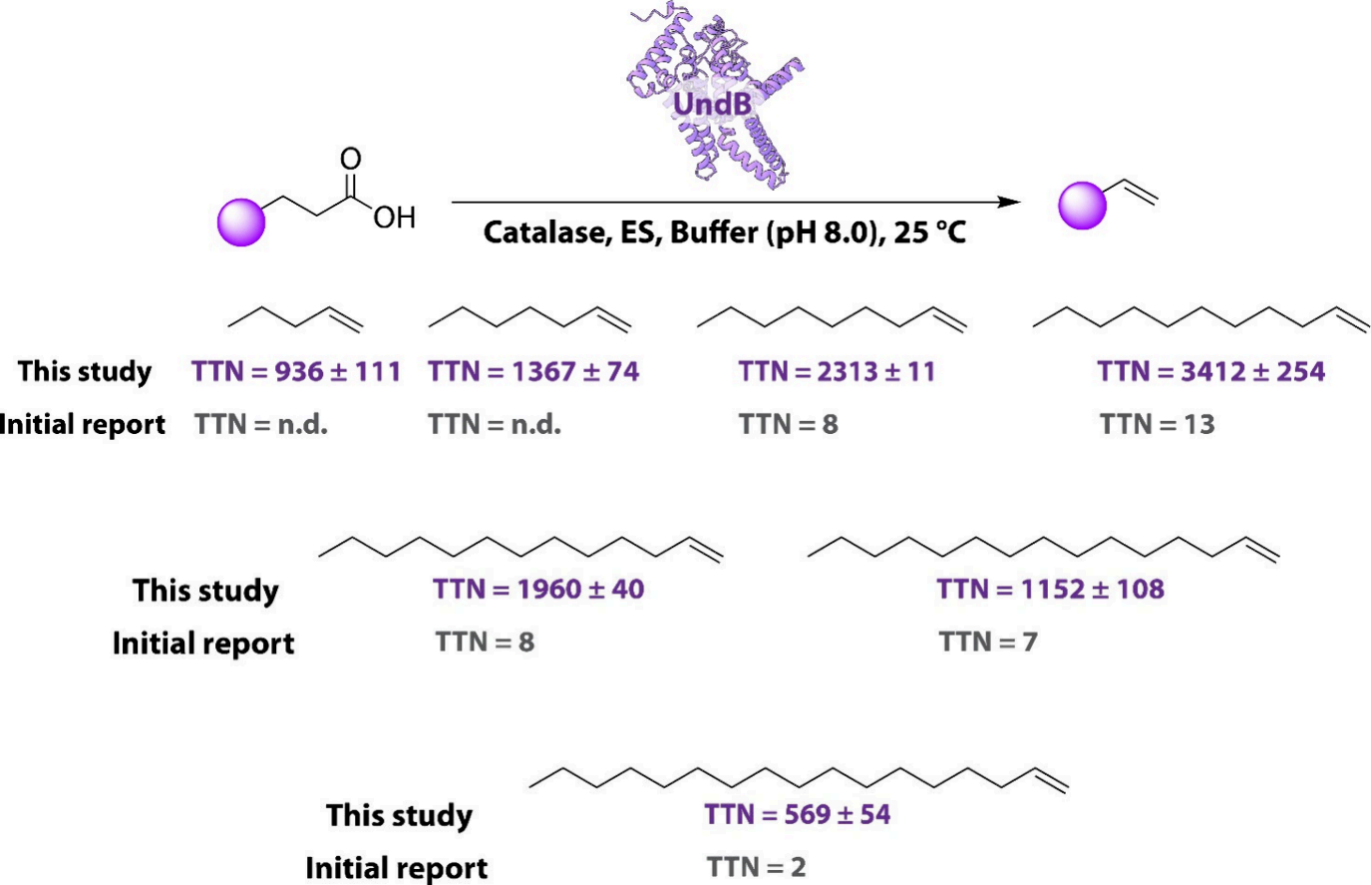
Production of 1-alkenes using an UndB-based CFB system. TTN of the UndB with various fatty acids using the CFB system developed in this study and initially reported TTN of UndB in the absence of any cofactor recycling system.^16^ All the experiments were performed in triplicate (*n* = 3), and the values report mean ± standard deviation. The bar graphs of each reaction performed at 25 and 4 °C, presenting the mean values and standard deviations, are presented in Figure S3.

### Structural Diversity Exploration Revealed a Key Region Responsible for Substrate Specificity of UndB

Substrate scope analysis of *Pme*-UndB revealed a higher production of 1-alkenes from medium-chain fatty acids. While lauric acid (C12) was efficiently converted to 1-undecene with a TTN of 3412 ± 254, the enzyme displayed significantly lower activity with long-chain substrates. For example, the reaction with palmitic acid (C16) yielded 1-pentadecene with a TTN of 1152 ± 108, which is nearly 3-fold lower than that of 1-undecene production from lauric acid (C12) (Figure 3). Given the greater natural abundance of long-chain fatty acids,^31,32^ we sought to identify UndB homologues with higher activity toward these substrates. The aim was to develop an UndB-based biocatalytic system for efficient production of high-value long-chain 1-alkenes from biobased feedstocks.^13^ We generated the structural models of 14 identified UndB homologues^15,16^ from diverse bacterial species using AlphaFold2.^33,34^ Our analysis revealed that UndB homologues presumably contain conserved global architecture among all homologues, characterized by four transmembrane helices (TMH1–TMH4) and a cytosolic domain consisting of six amphipathic helices (AH3, AH4, AH7-AH9, AH11) and nine soluble helices (H1, H2, H5, H6, H10, H12–H15) (Figure 4A) (Figure S4). We found that in all cases, conserved histidine residues were present within the cytosolic domain to coordinate the nonheme diiron catalytic center of the enzyme. However, subtle variation in the orientation of amphipathic helices AH8 and AH9 preceding TMH3 was observed (Figure 4A and 4B). Based on this structural disparity, UndB homologues can be classified into two classes: Class I, featuring extended AH8 and AH9 helices prior to TMH3 (present in bacterial families of *Pseudomonas*, *Acinetobacter*, and *Turneriella*, with sequence identities ranging from 36% to 77% with respect to *Pme*-UndB), and Class II, exhibiting a bent conformation of these helices in the same region (present in bacterial families of *Paraglaciecola*, *Burkholderia*, *Nocardia*, and *Leptospira*, with sequence identities ranging from 37% to 50% with respect to *Pme*-UndB) (Figure 4A and 4B) (Table S2).

**Figure 4:**
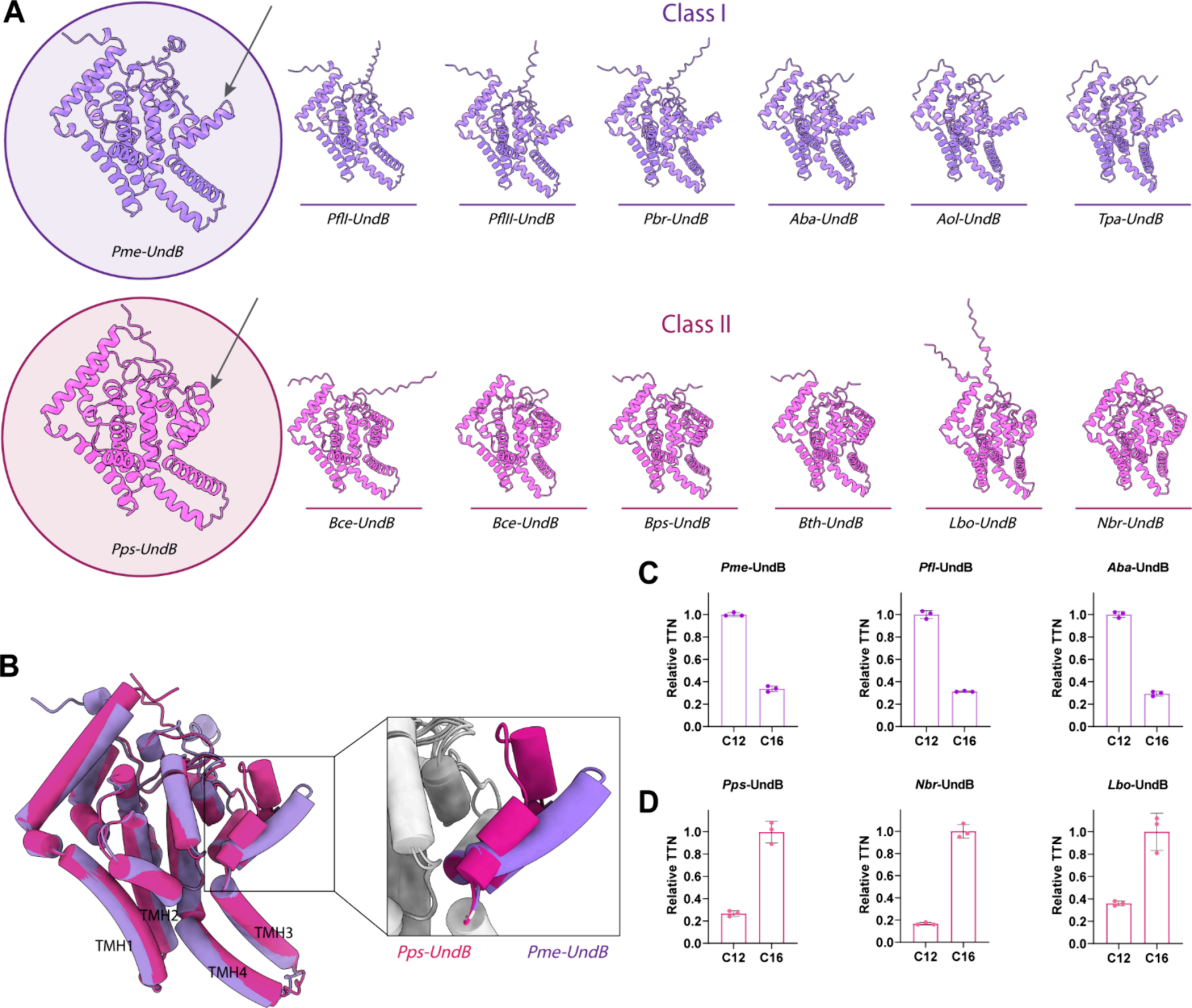
Structural diversity and relative activity of UndB homologues. (A) Modeled structures of the Class I (violet) and Class II (pink) UndB homologues obtained using AlphaFold2.^33,34^ Representative UndB of Class I (from *Pseudomonas mendocina* strain ymp; *Pme*-UndB) and Class II (from *Paraglaciecola psychrophile* strain 170; *Pps*-UndB) are shown in enlarged view for clear depiction. The key distal regions influencing substrate specificity of Class I and Class II homologues of UndB are depicted with arrows. (B) Overlaid modeled structures of *Pme*-UndB (violet) and *Pps*-UndB (pink). Both enzymes exhibit an overall similar structure except for the region highlighted in the zoomed-in image. Relative 1-alkenes production from lauric acid (C12) and palmitic acid (C16) by whole cells expressing (C) *Pme*-UndB, *Pfl*-UndB (from *Pseudomonas fluorescens* strain Pf-5) and *Aba*-UndB (from *Acinetobacter baumannii* strain *AYE*), and (D) *Pps*-UndB, *Nbr*-UndB (from *Nocardia brasiliensis*) and *Lbo*-UndB (from *Leptospira borgpetersenii*). Data reported in Figure 4C and Figure 4D are normalized to the 1-undecene and 1-pentadecene formed (taken as 1.0), respectively. Error bars represent the standard deviation (SD) of triplicate data (*n* = 3). The names of the bacterial species and the protein sequences of all the homologues are mentioned in the Supporting Information.

To assess the impact of this structural difference on substrate specificity, we compared the activity of the representative UndB enzymes from each class. We use *E. coli* whole cells expressing either *Pme*-UndB (Class I) or *Pps*-UndB (Class II; gene locus tag *C427_4391* from *Paraglaciecola psychrophile* strain 170) for activity analysis. Our assays demonstrated that *Pme*-UndB-expressing cells preferred lauric acid over palmitic acid, aligning with previous findings (Figure 4C). Interestingly, *Pps*-UndB-expressing cells showed a higher production of 1-alkenes from palmitic acid than from lauric acid, demonstrating a shift in substrate specificity (Figure 4D). This observation highlights how even a subtle structural difference distal (corresponding to residues 158–189 in *Pme*-UndB and residues 154–184 in *Pps*-UndB) to the diiron active site can significantly alter the substrate selectivity of the enzyme (Figure S5).

To further validate these findings, we analyzed two additional, distantly related homologues from each of Class I and Class II: *Pfl*-UndB (gene locus tag *Pfl_0203* from *Pseudomonas fluorescens* Pf-5) and *Aba*-UndB (gene locus tag *LV35_03289* from *Acinetobacter baumannii* AYE) from Class I and *Nbr*-UndB (gene locus tag *O3I_006310* from *Nocardia brasiliensis* strain ATCC700358) and *Lbo*-UndB (gene locus tag *LEP1GSC016_2993* from *Leptospira borgpetersenii* strain sv. Hardjo-bovis) from Class II (Figure 4C and 4D). Both Class I homologues (75%/85% and 60%/74% identity/similarity with *Pme*-UndB, respectively; Table S2) displayed strikingly higher alkene production from medium-chain lauric acid over long-chain palmitic acid, while both Class II homologues (47%/61% and 36%/55% identity/similarity with *Pme*-UndB; Table S2) showed higher alkene production from long-chain fatty acid, reinforcing the role of the distal region in determining substrate selectivity of UndB.

### Structure-Guided Enzyme Engineering Enabled UndB to Produce Long-Chain 1-Alkene Efficiently

Next, we sought to develop a CFB system with greater activity with long-chain fatty acids to produce high-value long-chain 1-alkenes. We hypothesized that swapping the postulated substrate selectivity region in *Pme*-UndB with the corresponding residues from *Pps*-UndB would generate an engineered UndB construct with greater activity with long-chain fatty acids. To test this, we engineered *Pme*-UndB (designated as ε*Pme*-UndB) by replacing residues 158–189 with residues 154–184 of *Pps*-UndB (Figure 5A).

**Figure 5:**
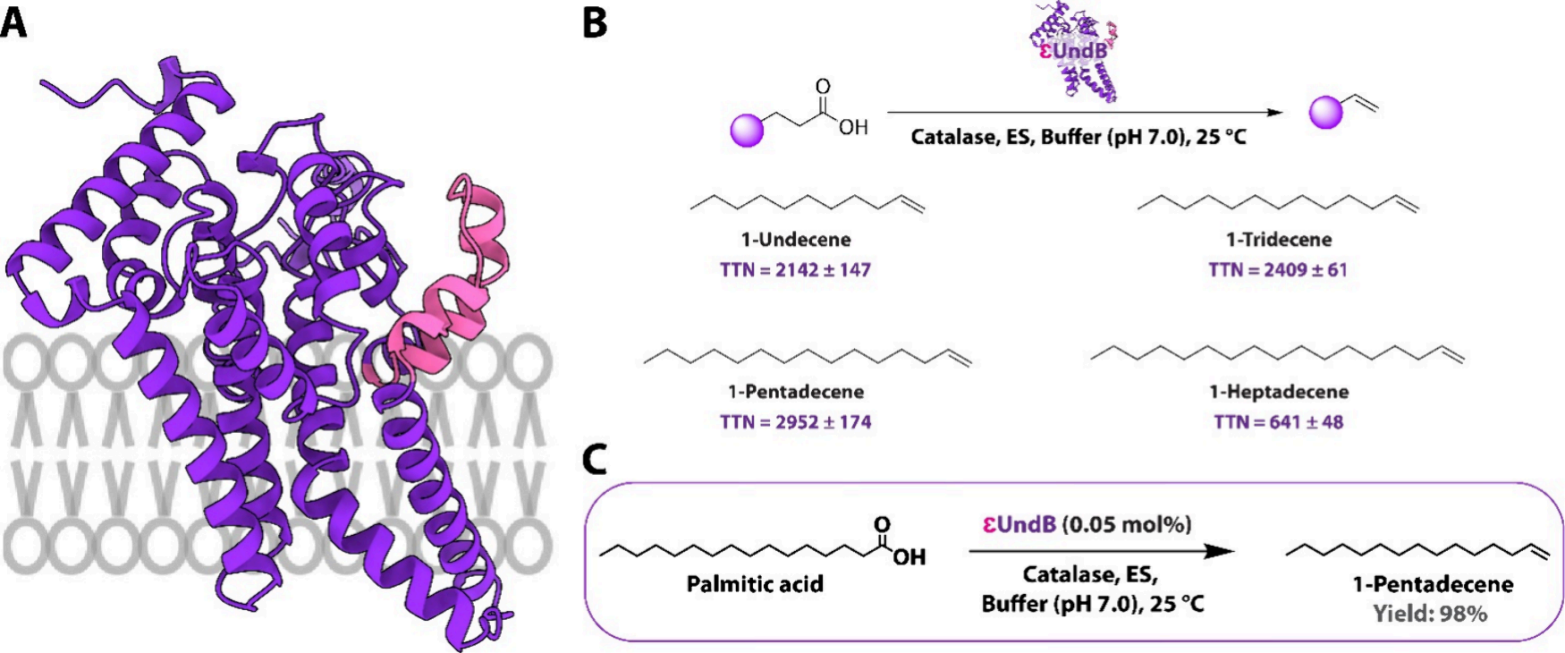
Structure and activity analysis of the engineered ε*Pme*-UndB. (A) Structure of the ε*Pme*-UndB obtained using Alphafold2.^33,34^ Secondary structure consisting of residues 158–189 highlighted in pink. (B) Activity analysis of ε*Pme*-UndB obtained with various fatty acids (at 1 mM concentration). (C) Schematic of the quantitative conversion of palmitic acid to 1-pentadecene using an engineered ε*Pme*-UndB-based biocatalytic system. All the experiments were performed in triplicate (*n* = 3), and the values report mean ± standard deviation. The bar graphs of each reaction performed in triplicate (*n* = 3) representing the mean values and standard deviations are presented in Figure S11. Comparative activities of *Pme*-UndB, ε*Pme*-UndB, and *Pps*-UndB are presented in Figure S12.

The engineered enzyme, ε*Pme*-UndB, displayed predicted structural characteristics aligning more closely with those of Class II UndB (Figure 5A). Subsequently, we measured the activity of ε*Pme*-UndB, which exhibited a substantial 2.6-fold increase in 1-pentadecene production (TTN = 2952 ± 254) (Figure 5B) compared to *Pme*-UndB (TTN = 1152 ± 108) (Figure 3). We also observed enhancements in the production of 1-tridecene and 1-heptadecene with our engineered UndB. We attributed this improved performance to the region-swapping engineering of UndB, and the enzyme, when integrated with our CFB platform, shifted its substrate specificity toward longer-chain fatty acids. This resulted in a significant improvement in the production of long-chain 1-alkenes, particularly 1-pentadecene. Importantly, the ε*Pme*-UndB-based cell-free platform resulted in a remarkable 98% yield of 1-pentadecene using a minimal catalyst loading of 0.05% under mild conditions (25 °C, pH 7.0).

To further probe the role of the putative substrate selectivity region, residues 158–189 from *Pme*-UndB were introduced into *Pps*-UndB, generating the engineered variant ε*Pps*-UndB. While this enzyme had the ability to convert lauric acid to undecene, it failed to produce detectable alkenes from long-chain fatty acids. Moreover, ε*Pps*-UndB exhibited a significant reduction in overall catalytic activity, likely due to the inherent instability of *Pps*-UndB, which displayed lower expression levels, reduced stability, and weaker catalytic performance compared to *Pme*-UndB. To expand this analysis, we performed a similar region swap in another Class II UndB homologue from *Nocardia brasiliensis* (strain ATCC700358), introducing *Pme*-UndB residues 158–189 to produce ε*Nbr*-UndB. This engineered variant displayed a substantial shift in substrate preference, favoring medium-chain fatty acids while showing minimal conversion of long-chain substrates (Figure S13). This activity profile more closely mirrored that of *Pme*-UndB rather than the native long-chain specificity typical of Class II homologues. These results strongly support the critical role of residues 158–189 in *Pme*-UndB in determining substrate chain-length specificity.

### Deciphering the Molecular Basis of Substrate Specificity of UndB

To investigate the substrate specificity of *Pme*-UndB and ε*Pme*-UndB, we conducted all-atom classical molecular dynamics simulations in the presence of a lipid bilayer (Figure 6A). A total of five simulations were performed, including *Pme*-UndB bound to lauric acid and palmitic acid, respectively, as well as ε*Pme*-UndB bound to the same substrates. Additionally, we carried out a simulation of *Pme*-UndB in its substrate-free form, i.e., in the absence of any bound substrate (Figures S6 and S7 and Table S1). Given the inherent challenges in capturing dynamic substrate–enzyme interactions in membrane proteins such as UndB using experimental methods, we envisioned that these simulations could offer critical atomistic insights into substrate binding.

**Figure 6:**
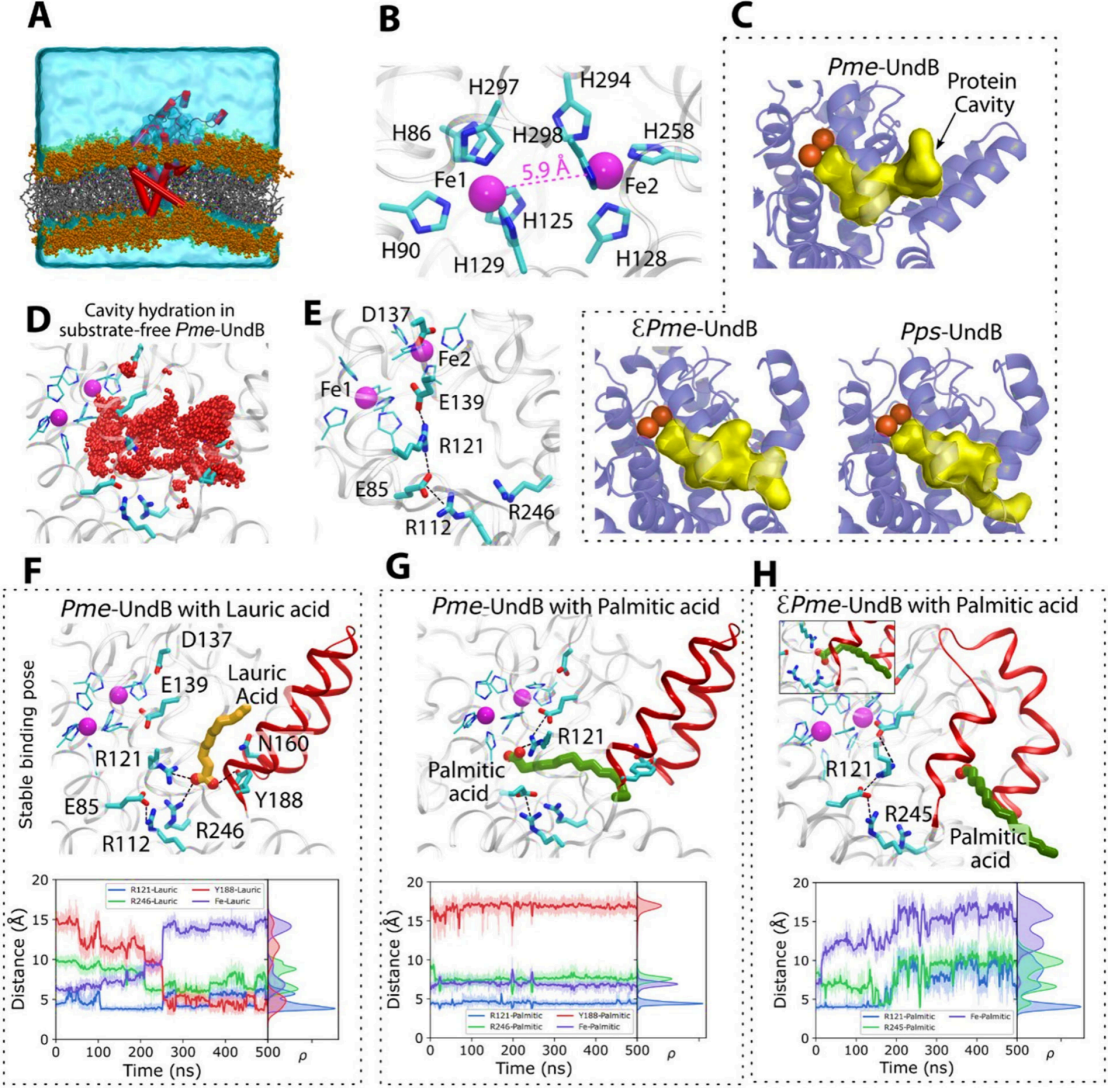
Molecular dynamics simulations of substrate–UndB interaction. (A) MD simulation setup with UndB embedded in a lipid bilayer and surrounded by a water box. The lipid bilayer consists of POPE (1-palmitoyl-2-oleoyl-*sn*-glycero-3-phosphoethanolamine) and POPG (1-palmitoyl-2-oleoyl-*sn*-glycero-3-(phospho-*rac*-(1-glycerol)). (B) Atomistic details of the metal–ligand architecture predicted by AlphaFold3, showing both iron atoms, nine coordinating histidine residues, and the Fe–Fe distance. (C) Representation of the substrate cavity volume (depicted as a yellow surface) for *Pme*-UndB (∼700 Å^3^), ε*Pme*-UndB (∼964 Å^3^), and *Pps*-UndB (∼987 Å^3^) predicted using the Fpocket suite.^38^ (D) Hydration of the substrate-binding cavity in the MD simulations of substrate-free *Pme*-UndB (without a substrate). The red spheres depict the oxygen atoms of water molecules. (E) Network of charged residues extending from the diiron site to the high-affinity binding site. (F) Stable substrate-binding site for lauric acid (shown in yellow color) in *Pme*-UndB from MD simulations. (G) Stable substrate-binding site for palmitic acid (shown in green color) in *Pme*-UndB. (H) Stable substrate-binding site for palmitic acid (shown in green color) in ε*Pme*-UndB. The secondary structure comprising residues 158–189 is highlighted in red. Inset shows a snapshot from MD simulations showing palmitic acid interacting with R121 and R245 before diffusing into the membrane. Corresponding distance evolution between the diiron site, key residues (R121, Y188, and R246/R245), and the substrate from MD simulations are shown below the respective substrate-binding poses (Movies S1–S4).

For both *Pme*-UndB and ε*Pme*-UndB, we predicted their ligand-bound three-dimensional model structures using AlphaFold3 (AF3).^35^ AF3 predicted the Fe1 and Fe2 centers to be separated by 5.9 Å, with Fe1 coordinated by five histidine residues and Fe2 by four (Figure 6B). Notably, this metal–ligand architecture closely resembles that of mammalian stearoyl-CoA desaturase, which exhibits a metal–metal distance of 6.4–6.7 Å.^36,37^ Interestingly, AF3 predicted the substrate to coordinate the Fe2 center, with the fatty acid tail extending through the diiron pocket toward the bulk solvent (Figure S6B). This configuration served as the starting point for all MD simulations in this study. The AF3-predicted substrate binding location is consistent with the cavity volume of the substrate-free forms of *Pme*-UndB, ε*Pme*-UndB, and *Pps*-UndB. Specifically, ε*Pme*-UndB and *Pps*-UndB are predicted to have significantly larger pocket volumes (Figure 6C and 6D), aligning with their higher specificity for palmitic acid compared to that of *Pme*-UndB.

In the case of *Pme*-UndB bound to lauric acid, our MD simulations suggest that the high-affinity binding site for the substrate is located approximately 14 Å from the diiron center (Figure 6E and 6F, Movie S1). At this site, the carboxylate group of the fatty acid forms an ion-pair network with R121, R246, and E85 and establishes a hydrogen bond interaction with Y188. Interestingly, for *Pme*-UndB bound to palmitic acid, we observed that the substrate remains highly stable, within the time scale of our simulations, near the diiron center, forming a strong ion-pair interaction with R121 (Figure 6G, Movie S2). The differences in the binding modes of these substrates may be attributed to their chain lengths and cavity volume, which likely influence their conformational flexibility. For example, our MD simulations of ε*Pme*-UndB with palmitic acid revealed that the substrate binds initially at the high-affinity site before diffusing into the lower leaflet of the lipid bilayer (Movie S3). Notably, Y188 in *Pme*-UndB is replaced by S187 in ε*Pme*-UndB (with N160 replaced by G160), which not only alters the binding interactions but also enlarges the cavity volume, creating a further connection with the lipid bilayer (Figure 6E). These results clearly indicate that the substitution of the 158–189 region in *Pme*-UndB modulates the substrate-binding cavity volume, thereby influencing the substrate binding. Interestingly, despite the larger cavity volume, our MD simulations of ε*Pme*-UndB with lauric acid reveal a binding pose at the high-affinity site similar to that observed in *Pme*-UndB (Figure S8 and Movie S4). We also note that our results remain consistent when changing the POPE:POPG membrane composition from 4:1 to 7:3, which overall supports the possible role of the 158–189 region in regulating substrate recognition and dynamics (Figure S9).

Our results clearly demonstrate that ion-pair networks play a pivotal role in substrate binding and stabilization. We identified a substantial network of ion-pair interactions and charged residues that facilitate this process including D137, E139, R121, E85, R112, and R246 (Figure 6E). Notably, the E139, R121, E85, and R246 residues are particularly critical for substrate binding. This finding is further supported by our experimental analysis of the R121F mutant, which showed drastically reduced activity (<10%) (Figure S14). Recent mutagenesis experiments further support our observations, showing that alanine-scanning mutants of E139A and R121A drastically reduced the production of 1,7-octadiene.^39^ While these residues help maintain a rigid network within the substrate-binding cavity, we also observed that R121 exhibits significant flexibility. This flexibility enables it to stabilize ligands in both the proximal and distal binding sites relative to the diiron cluster (Figure 6G and 6H).

We also observed that water molecules play a crucial role in the dynamics of the UndB–substrate complex. In our substrate-free *Pme*-UndB simulations, the substrate cavity was found to be fully occupied by water molecules (Figure 6D), forming a continuous channel from the bulk solvent to the diiron site. Interestingly, upon substrate binding, water accessibility to the diiron site was significantly reduced, although not entirely blocked (Figure S10).

Overall, our atomistic simulations suggest that substrate specificity arises from the volumetric modulation of the binding pocket. Region-swapping in *Pme*-UndB increases the substrate-binding pocket volume, conferring specificity to ε*Pme*-UndB for palmitic acid, while *Pme*-UndB, with a slightly smaller pocket volume, shows higher specificity for lauric acid. Additionally, we identified a distal high-affinity substrate-binding site and a network of ion-pair interactions extending from the di-iron catalytic site to the high-affinity site. Notably, R121, a highly dynamic and central residue in this ion-pair network, plays a crucial role in stabilizing the substrate both at the high-affinity site and at the binding site near the diiron center. Based on our simulations, however, it remains challenging to elucidate what conformational changes occur upon O_2_ activation that may facilitate the movement of the fatty acid from the high-affinity binding site to a more proximal position conducive to catalysis. The role of water in the catalytic process also warrants further investigation. Future structural studies of UndB in native-like environment, such as lipid nanodiscs, may further facilitate detailed investigations.^40–43^ Nevertheless, our studies provide a platform to investigate further how substrate binding at the high-affinity site influences the redox properties of the diiron center and whether it facilitates the opening of a transient gas channel for O_2_ entry, as observed in toluene/*o*-xylene monooxygenase hydroxylase.^44^

## Conclusion

This study marks a significant advancement in developing an efficient cell-free biocatalytic platform utilizing UndB for the bioproduction of high-value 1-alkenes from inexpensive and naturally abundant free fatty acids. First, by integrating UndB with two co-substrate recycling systems coupled with inhibitor metabolism, we have developed a simple yet highly effective biocatalytic system achieving remarkable activity in converting lauric acid into 1-undecene with an unprecedented TTN of 3412 ± 254. This marks a 262-fold improvement over initially reported UndB activity,^16^ highlighting the potential of the biocatalytic system as a green alternative to traditional, chemically harsh methods for 1-alkene production. Our approach not only enhanced the overall activity of UndB but also reduced reliance on large quantities of expensive cosubstrate, NADPH, making the process more cost-effective.

More importantly, we explored the predicted model-based structural features of UndB homologues and identified a crucial structural region responsible for substrate selectivity. Through region-swapping engineering, we experimentally validated the pivotal role of this segment in determining the substrate chain-length specificity. Thereafter, employing structure-guided enzyme engineering, we developed a state-of-the-art UndB-based system favoring naturally more abundant long-chain fatty acids and leading to the production of the corresponding 1-alkenes. Furthermore, our studies offer insights into the molecular basis of substrate specificity, driven by volumetric changes in the substrate-binding pocket of UndB. In particular, we highlighted the role of key residues in forming an ion-pair network, potentially stabilizing the substrate in both distal and proximal positions relative to the diiron site. The cell-free biocatalytic systems developed, using native *Pme*-UndB and engineered ε*Pme*-UndB, achieved 98% yield in the production of 1-undecene and 1-pentadecene, respectively, with extremely low catalyst loading (0.04 mol % and 0.05 mol %, respectively) under mild conditions. To the best of our knowledge, this is the first report of engineering a cell-free biocatalytic platform using an integral membrane metalloenzyme for high-yield hydrocarbon production. These cell-free biocatalytic systems, developed using UndB, also hold promise for applications in biocatalytic cascades and the development of new chemoenzymatic methods for producing medicinally and industrially important cycloalkenes,^45,46^ amines,^47–51^ cyclopropanes,^52,53^ and esters.^54^

## Experimental Section

### Protein Expression, Purification, and CEF Isolation

Methods for the expression and purification of *Pme*-UndB, KatE-UndB, ferredoxin, and ferredoxin reductase, bacterial growth, and CEF isolation are provided in the Supporting Information. No unexpected or unusually high safety hazards were encountered.

### Bioinformatics, Structural Modeling, and MD Simulations

All sequences are retrieved from the IMG/JGI database (https://img.jgi.doe.gov/cgi-bin/w/main.cgi), and the sequences are listed in the Supporting Information. AlphaFold2 was used for generating the structural models of UndB homologues, while AlphaFold3 was used to predict the diiron- and substrate-bound structures.^35^ All protein–substrate models were embedded in a lipid bilayer and surrounded by a water box. Systematic minimization and equilibration steps were performed prior to production simulations, which were conducted in the *NPT* ensemble at *T* = 303 K and 1 atm, with an integration time step of 2 fs. Each UndB–substrate model was simulated for 500 ns, while the substrate-free model of *Pme*-UndB was simulated for 250 ns, resulting in a cumulative simulation time of 2.25 μs. All MD simulations were carried out using the AMBER24 suite.^55^ A detailed description of the system setup, molecular mechanics parametrization, and MD simulations is provided in the Supporting Information.

### General Procedure for 1-Alkene Production

Reactions were performed in 1.5 mL gas chromatography vials, and the typical reaction volume was 0.5 mL. A standard reaction mixture contained the following components at final concentrations unless otherwise mentioned: 0.1 μM UndB or KatE-UndB (purified or present within CEF), 1 mg/mL catalase (catalog no. C9322, Sigma-Aldrich, USA), 2.5 μM ferredoxin reductase, 15 μM ferredoxin, 1 mM fatty acid, 10 U/mL glucose dehydrogenase (Catalog no. 19359, Sigma-Aldrich), 2 mM d-glucose, and 200 μM NADPH. The assays were performed in assay buffer [50 mM HEPES (pH 8.0) containing 200 mM NaCl, 0.005% (w/v) LMNG, 100 μM Fe (NH_4_)_2_Fe­(SO_4_)_2_·6H_2_O, and 1 mM CaCl_2_] at 25 or 4 °C for the indicated time points. The reactions also contained 0.00025% (v/v) tergitol NP-10, 5% (v/v) DMSO, or 5% (v/v) ethanol as cosolvents, as indicated in the text. The assays were initiated with the addition of NADPH, and the products were analyzed by GC-MS as described in the Supporting Information.

## Supplementary Material











## Notes

Materials, gene sequences, methods, computational details, supporting figures and tables (PDF)
Movie S1: MD simulation movie (MP4)
Movie S2: MD simulation movie (MP4)
Movie S3: MD simulation movie (MP4)
Movie S4: MD simulation movie (MP4)

## ABBREVIATIONS

TTN: total turnover number
MD: molecular dynamics
GDH: glucose dehydrogenase
CEF: cellular membrane envelope fractions
*Pme*-UndB: UndB from *Pseudomonas mendocina* ymp
*Pps*-UndB: UndB from *Paraglaciecola psychrophile* 170
ε*Pme*-UndB: engineered *Pme*-UndB
