# Supplementary material for: Engineering of Integral Membrane Metalloenzyme UndB and Designing of a Cell-Free Biocatalytic Platform Enabled Efficient 1‑Alkene Production
Source: ACS Cent Sci. 2025 Oct 14;11(12):2329–39. doi: 10.1021/acscentsci.5c01099 (PMC12746148; doi:10.1021/acscentsci.5c01099)
Supplement: Supplementary file 1 [file oc5c01099_si_001.pdf]

Supporting Information

Engineering of Integral Membrane Metalloenzyme UndB and Designing of a Cell-Free Biocatalytic Platform Enabled Efficient 1-Alkene Production

Tabish Iqbal[1†], Subhashini Murugan[1†], Jayaprakash Karupusamy[1], Abhishek Sirohiwal[1*], and Debasis Das[1*]

[1]*Department of Inorganic and Physical Chemistry, Indian Institute of Science, Bangalore, Karnataka-560012, India*

†These authors contributed equally

***Corresponding Authors**: Debasis Das and Abhishek Sirohiwal

Phone number: +91-8022933002

Email: debasisdas@iisc.ac.in and asirohiwal@iisc.ac.in

Supporting Information Text

Materials

Lysogeny broth (LB, catalog no. M1245), terrific broth (TB, catalog no. M1250) and agar, Type 1 (GRM666) for bacterial culture were obtained from HiMedia, India. The detergent n-lauryl maltose neopentyl glycol (LMNG, catalog no. NG310) was purchased from Anatrace, USA. Lysozyme (catalog no. 210083125) was obtained from MP Biomedical, USA. DNaseI (catalog no. RC1072) was obtained from G-Bioscience, USA. Kanamycin monosulphate (catalog no. 99311), ampicillin (catalog no. 61314), GC-MS grade ethyl acetate (catalog no. 73106), NADPH (β-nicotinamide adenine dinucleotide phosphate tetrasodium salt (reduced) (catalog no. 99197), 4-(2-hydroxyethyl)-1-piperazineethanesulfonic acid (HEPES, catalog no. 16826), tris(hydroxymethyl)aminomethane (Tris), catalog no. 71033) glycerol (catalog no. 59991), 5-aminolevulinic acid hydrochloride (catalog no. 33864), and salts with the purest commercial grade were obtained from SRL Chemicals, India. Ammonium iron (II) sulfate hexahydrate ($(NH_4)_2Fe(SO_4)_2.6H_2O$)) (catalog no. 203505), bovine liver catalase (catalog no. C9322), glucose dehydrogenase from *Pseudomonas sp*. (catalog no.19359), glucose (catalog no. G8270) and tergitol NP-10 (NP-10, catalog no. T1135) were purchased from Sigma Aldrich, USA. FAD (flavin adenine dinucleotide disodium salt hydrate, catalog no. F0014), and all fatty acids (hexanoic acid (catalog no. H0105), octanoic acid (catalog no. O0027), decanoic acid (catalog no. D0017), lauric acid (catalog no. L0011), tetradecanoic acid (catalog no. M0476), hexadecenoic acid (catalog no. P1145), octadecanoic acid (catalog no. S0163)) and 1-alkenes (1-pentene (catalog no. P0316), 1-heptene (catalog no. S0337), 1-nonene (catalog no. S0339), 1-decene (catalog no. D0028), 1-undecene (catalog no. U0025), 1-tridecene (catalog no. T0637), 1-pentadecene (catalog no. S0345), 1-heptadecene (catalog no. S0347)) standards were obtained from TCI chemical, Japan. Nickel-nitrilotriacetic acid (Ni-NTA) resin (catalog no. 1560137) was purchased from BioRad, USA. All pre-packed columns and cartridges were obtained commercially. All the other chemicals and biochemicals were of the highest available commercial grades.

Gene constructs design

The full-length, *Pmen_4370* gene (UniProt ID: A4Y0K1), *C427_4391* gene (UniProt ID: K7AEV5), *PFL_0203* gene (UniProt ID: Q488K4), *LV35_03289* gene (UniProt ID: A0A0D7UIY5), *O3I_006310* gene (UniProt ID: K0ER28) and *LEP1GSC016_2993* gene (UniProt ID: M6BK47) encoding UndB from *Pseudomonas mendocina* (strain *ymp*), *Paraglaciecola psychrophile* 170, *Pseudomonas fluorescens* Pf-5, *Acinetobacter baumannii* AYE, *Nocardia brasiliensis* strain ATCC700358 and *Leptospira borgpetersenii* strain sv. Hardjo-bovis respectively, were codon optimized for expression in *E. coli* and synthesized (GenScript, USA). The *εPme-UndB* gene was constructed by replacing base pairs 472-567 (residues 158-189) from *Pmen_4370* gene with base pairs 460-552 (residues of 154-184) of *C427_4391* gene, *εPps-UndB* gene was constructed by replacing base pairs 460-552 (residues 154-184) from *C427_4391* gene with base pairs 472-567 (residues of 158-189) of *Pmen_4370* gene and *εNbr-UndB* gene was constructed by replacing base pairs 490-588 (residues 166-207) from *O3I_006310 gene from Nocardia brasiliensis strain (ATCC700358)* with base pairs 472-567 (residues of 158-189) of *Pmen_4370* gene. The *Pmen_4370*, *C427_4391*, *Pfl_0203* , *LV35_03289*, *O3I_006310*, *LEP1GSC016_2993*, *εPme-UndB, εPps-UndB and εNbr-UndB* genes were cloned with a C-terminal flexible linker (SSG), and the TEV protease site into the pET-16b(+) vector harboring a gene encoding eGFP-His_8 using the restriction site NcoI and NotI to generate the *Pme*-UndB-TEV-eGFP-His_8, *Pps*-UndB-TEV-eGFP-His_8, *Pfl*-UndB-TEV-eGFP-His_8, *Aba*-UndB-TEV-eGFP-His_8, *Nbr*-UndB-TEV-eGFP-His_8, *Lbo*-UndB-TEV-eGFP-His_8, ε*Pme*-UndB-TEV-eGFP-His_8, ε*Pps*-UndB-TEV-eGFP-His_8 and ε*Nbr*-UndB-TEV-eGFP-His_8 The *KatE-UndB* gene was

constructed by cloning *KatE* gene of *E. coli* (UniProt ID: P21179) encoding catalase HPII into a pET-16b(+) vector, followed by a SSG linker, followed by the *Pmen_4370* gene, followed by the sequence encoding for 8*His-tag. The initial 24 nucleotides were deleted from the UndB encoding gene to remove the first 8 residues, resulting construct KatE-SSG-UndB-His_8 (KatE-UndB) was codon optimised for expression in *E. coli* and synthesized (GenScript, USA).

Protein sequences of the constructs used in this study

Pme-UndB (_Pme_-UndB-SSG-TEV-eGFP- His$_8$)

Pme-UndB: Full length *Pmen_4370* gene from *P. mendocina* (*strain ymp*) (UniProt ID: A4Y0K1)

SSG: Three residue long linker (Ser-Ser-Gly)

TEV: TEV-protease cleavage site

eGFP: enhanced green fluorescent protein

His$_8$: 8xHis-tag

MEMSPSPASLNDQQRAAHIREQVMAHGNALRQRYPILQHQDALGAGILAFALCGMIGSAALYIGGHLPW
WACLLLNAFFASLTHELEHDLIHSMYFRKQPLPHNLMLALVWLARPSTINPWVRRHLHLNHHKVSGSE
ADMEERAITNGEPWGIARLLMVGDNMMSSFIRWLRAKNPEHRRLILTRTLKVYAPLGLLNWATWYLFL
GFHLLDWAAAALGAPIAWSASTLSVMQVVNVAVVVLVGPNVLRTFCLHFVSSNMHYYGDVEPGNVIQQ
TQVLNPWWLWPLQAFCFNFGSSHAIHHFVVKEPFYIRQLTVPFAHRVMREMGVRFNDFGTFARANRW
TRRARTQQERASTASSGTENLYFQSGAAAAVSKGEELFTGVVPILVELDGDVNGHKFSVSGEGEGDATYG
KLTLKFICTTGKLPVPWPTLVTTLTYGVQCFSRYPDHMKQHDFFKSAMPEGYVQERTIFFKDDGNYKTR
AEVKFEGDTLVNRIELKGIDFKEDGNILGHKLEYNYNSHNVYIMADKQKNGIKVNFKIRHNIEDGSVQLA
DHYQQNTPIGDGPVLLPDNHYLSTQSKLSKDPNEKRDHMVLLEFVTAAGITLGMDELYKSGLRSHHHH
HHHH

KatE-UndB (KatE-SSG-UndB-His$_8$)

KatE- *KatE* gene of *Escherichia coli* (UniProt ID: P21179) encoding HPII catalase

SSG: Three-residues long linker (Ser-Ser-Gly)

UndB: *Pmen_4370* gene from *P. mendocina* (*strain ymp*) (Initial 24 nucleotides were deleted from the UndB encoding gene to remove the first 8 residues)

His$_8$: 8xHis-tag

MESQHNEKNPHQHQSPLHDSSEAKPGMDSLAPEDGSHRPAAEPTPPGAQPTAPGSLKAPDTRNEKLNS
LEDVRKGSENYALTTNQGVRIADDQNSLRAGSRGPTLLEDFILREKITHFDHERIPERIVHARGSAAHGYF
QPYKSLSDITKADFLSDPNKITPVFVRFSTVQGGAGSADTVRDIRGFATKFYTEEGIFDLVGNNTPIFFIQD
AHKFPDFVHAVKPEPHWAIPQGQSAHDTFWDYVSLQPETLHNVMWAMSDRGIPRSYRTMEGFGIHTF
RLINAEGKATFVRFHWKPLAGKASLVWDEAQKLTGRDPDFHRRELWEAIEAGDFPEYELGFQLIPEEDE
FKFDFDLLDPTKLIPEELVPVQRVGKMVLNRNPDNFFAENEQAAFHPGHIVPGLDFTNDPLLQGRLFSY
TDTQISRLGGPNFHEIPINRPTCPYHNFQRDGMHRMGIDTNPANYEPNSINDNWPRETPPGPKRGGFES
YQERVEGNKVRERSPSFGEYYSHPRLFWLSQTPFEQRHIVDGFSFELSKVVRPYIRERVVDQLAHIDLTL
AQAVAKNLGIELTDDQLNITPPPDVNGLKKDPSLSLYAIPDGDVKGRVVAILLNDEVRSADLLAILKALKA
KGVHAKLLYSRMGEVTADDGTVLPIAATFAGAPSLTVDAVIVPCGNIADIADNGDANYYLMEAYKHLKPI
ALAGDARKFKATIKIADQGEEGIVEADSADGSFMDELLTLMAAHRVWSRIPKIDKIPASSGNDQQRAAHI
REQVMAHGNALRQRYPILQHQDALGAGILAFALCGMIGSAALYIGGHLPWWACLLLNAFFASLTHELEH
DLIHSMYFRKQPLPHNLMLALVWLARPSTINPWVRRHLHLNHHKVSGSEADMEERAITNGEPWGIAR

LLMVGDNMMSSFIRWLRAKNPEHRRLILTRTLKVYAPLGLLNWATWYLFLGFHLLDWAAAALGAPIA
WSASTLSVMQVVNVAVVVLVGPNVLRTFCLHFVSSNMHYYGDVEPGNVIQQTQVLNPWWLWPLQAFC
FNFGSSHAIHHFVVKEPFYIRQLTVPFAHRVMREMGVRFNDFGTFARANRWTRRARTQQERASTASSGT
LYKSGLRSHHHHHHHH

***Pps*-UndB_GFP**

***Pps*-UndB:** Full length *C427_4391* gene encoding UndB from *P. mendocina* (strain *ymp*) (UniProt ID: K7AEV5)

SSG: Three residue long linker (Ser-Ser-Gly)

TEV: TEV-protease cleavage site

eGFP: enhanced Green fluoresenct protein

His$_8$: 8xHis-tag

MEMQNNQDKQDIKEIVAYIKNQERTLRSNHPFLAQQNALGLGLLLVSVCGFTAAGCLYFYAVIPAWCCIII
AALSASIAHEIEHDLIHQQYFKSNSAVYHFMMFMVWIIRPNTVNPWYRKGMHLNHHKTSGTPQDIEER
LVGNGIKSHTLRLLVVCDGLLGLIIRSKQFSREIKGYNFFNVFNASLPFVTVYYLIIYIFLLFHGVNFIADSSA
VKLNTPVWLVSLMEWVNFAMVVWVAPNFLRSACLNFITSSMHYYGARFNLLEQTQVLNHWAFMPFQ
WFCFNFGHTHSIHHFVPNQPFYIRQIISKQVNLLLKNKGVKFNDLVSIFAANHYKKIERSSSSGTENLYFQ
SGAAAAVSKGEELFTGVVPILVELDGDVNGHKFSVSGEGEGDATYGKLTLKFICTTGKLPVPWPTLVTTLT
YGVQCFSRYPDHMKQHDFFKSAMPEGYVQERTIFFKDDGNYKTRAEVKFEGDTLVNRIELKGIDFKEDG
NILGHKLEYNYNSHNVYIMADKQKNGIKVNFKIRHNIEDGSVQLADHYQQNTPIGDGPVLLPDNHYLST
QSKLSKDPNEKRDHMVLLEFVTAAGITLGMDELYKSGLRSHHHHHHHH

***Pfl*-UndB_GFP**

***Pfl*-UndB:** Full length *PFL_0203* gene encoding UndB from *Pseudomonas fluorescens* Pf-5 (UniProt ID: Q4KK84)

SSG: Three residue long linker (Ser-Ser-Gly)

TEV: TEV-protease cleavage site

eGFP: enhanced Green fluoresenct protein

His$_8$: 8xHis-tag

MEMQGISASPERMNAQQRAAHVRQVVLARGDELRRRFPLLRHQDALGAGILAFALSGMLGSALLYVTG
HLAWWACLLLNAFFASLTHELEHDLIHSMYFRKQRLPHNLMLGLVWLARPSTINPWVRRHLHLNHHK
VSGSESDIEERAITNGEPWGIARLLMVGDNMMAAFIRLLRAPGARRKLGILVRTLAVYAPLALLHWGAW
YVFLGFHGANGVAALLGSPIQWSQDTASLMHYVDIAVVVIIGPNVLRTFCLHFVSSNMHYYGDIEPGNVI
QQTQVLNPWWMWPLQAFCCNFGSTHGIHHFVVREPFYIRQMTASVAHKVMAEMGVRFNDFGTFARA
NRFTRQEREAMQPAHNARASSGTENLYFQSGAAAAVSKGEELFTGVVPILVELDGDVNGHKFSVSGEGE
GDATYGKLTLKFICTTGKLPVPWPTLVTTLTYGVQCFSRYPDHMKQHDFFKSAMPEGYVQERTIFFKDD
GNYKTRAEVKFEGDTLVNRIELKGIDFKEDGNILGHKLEYNYNSHNVYIMADKQKNGIKVNFKIRHNIE

DGSVQLADHYQQNTPIGDGPVLLPDNHYLSTQSKLSKDPNEKRDHMVLLEFVTAAGITLGMDELYKSGL
RSHHHHHHHH

***Aba*-UndB_GFP**

***Aba*-UndB**: Full length *LV35_03289* gene encoding UndB from *Acinetobacter baumannii* AYE (UniProt ID: A0A0D7UIY5)

SSG: Three residue long linker (Ser-Ser-Gly)

TEV: TEV-protease cleavage site

eGFP: enhanced Green fluoresenct protein

His$_8$: 8xHis-tag

MEMTYIYKNPAGMTDSEKTEHIKKVVTAEGVALRKRHPILNHQNAIGAMILFISLVGMIATAVLYINHQLS
AWFAIPIIAFFASLTHELEHDLIHWMYFRKKPWAHHLMMGLVWLARPSTINPWKRRELHFNHHKNSG
TEVDLEERALTNGEQWSIRRLIAIGDNGLAVLFRIISASNWTVRKVIFKRAFMAYFPLGIIHWSLWYIFLG
FHAVDAVLSWANAPIAWSATTLNIMHVVNILTVVWVAPNVLRTFCLHFVTSNMHYYGDVELGNVIQQT
QVLKPWWMMPFQLFCFNFGSTHAIHHFVVKEPFYIRQMTAPVAHKVMRDMGVRFNDVGTFKRANRW
NINDLSESKSSSGTENLYFQSGAAAAVSKGEELFTGVVPILVELDGDVNGHKFSVSGEGEGDATYGKLTLK
FICTTGKLPVPWPTLVTTLTYGVQCFSRYPDHMKQHDFFKSAMPEGYVQERTIFFKDDGNYKTRAEVKF
EGDTLVNRIELKGIDFKEDGNILGHKLEYNYNSHNVYIMADKQKNGIKVNFKIRHNIEDGSVQLADHYQ
QNTPIGDGPVLLPDNHYLSTQSKLSKDPNEKRDHMVLLEFVTAAGITLGMDELYKSGLRSHHHHHHHH

***Nbr*-UndB_GFP**

***Nbr*-UndB**: Full length *O3I_006310* gene encoding UndB from *Nocardia brasiliensis* strain ATCC700358 (UniProt ID: K0ER28)

SSG: Three residue long linker (Ser-Ser-Gly)

TEV: TEV-protease cleavage site

eGFP: enhanced Green fluoresenct protein

His$_8$: 8xHis-tag

MEMRLATRLPGERTLAPQDADRIAAIRGEIARVGDRWRVEHPWIAGHQNTIGAVIFLGAVLGVLGDAAL
YACGLLPWWATVLASAFWLSLLHEIEHDLIHAMYFRTNKWVHNAMLAGVWLLRPSTINPWVRRRLHL
HHHAVSGTESDLEERAISNGERWGGHRLLGLLDSVLGYATRPFRMRGLVAAYVARVARDPAEARRLAITT
PLAYFPLSAMHYGLWYLTVSAHVYELLGGTVGYPGAYRALDILAVTLLAPNAIRTFCLYFVSSNLHYYGDV
EPHNVLQQTQVWTARWLWPVHALCFNFGGTHAIHHFVVRDPFYIREAIRAECQTILREHGVRFNDFGT
FRRANRFGLAVPPGAVRPSSGTENLYFQSGAAAAVSKGEELFTGVVPILVELDGDVNGHKFSVSGEGEGD
ATYGKLTLKFICTTGKLPVPWPTLVTTLTYGVQCFSRYPDHMKQHDFFKSAMPEGYVQERTIFFKDDGN
YKTRAEVKFEGDTLVNRIELKGIDFKEDGNILGHKLEYNYNSHNVYIMADKQKNGIKVNFKIRHNIEDGS
VQLADHYQQNTPIGDGPVLLPDNHYLSTQSKLSKDPNEKRDHMVLLEFVTAAGITLGMDELYKSGLRSH
HHHHHHH

Lbo-UndB_GFP

Lbo-UndB: Full length _LEP1GSC016_2993_ gene encoding UndB from (UniProt ID: M6BK47)

SSG: Three residue long linker (Ser-Ser-Gly)

TEV: TEV-protease cleavage site

eGFP: enhanced Green fluoresenct protein

His$_8$: 8xHis-tag

MEMNSQWKTRNKPYKVFSEKEKTRKIIQWIRFWDDRIRNRFPYLSKYQDQIGFGIMIGSASGMILFAVL
YITNLIPFWFCIVLNTIFASFLHEIEHDLIHNLYYKGRVKVQNFMLWVVWLFRANTVNPWFRREIHLLH
HKLSGNKEDVEERMIGNGVPFGLKRVLIMIDGNLALILQGRKVAKDAYLRLGKIKVPRTVGLYRETFFLL
WYSFLSVNAFHILNVLFGNPISEPSFLETNRSVLNSAAVVYLIPNWIRQTSLQVVSSNMHYYGNVPNVYH
QTQVLNSWLVFPFHLFCFNFGATHGIHHFVVNQPFYLRQWVAFYVLSAMKRYGIRFNDFRSMWKSNSE
SLLEENKIDFSKMTNFPISNSKSSGTENLYFQSGAAAAVSKGEELFTGVVPILVELDGDVNGHKFSVSGEG
EGDATYGKLTLKFICTTGKLPVPWPTLVTTLTYGVQCFSRYPDHMKQHDFFKSAMPEGYVQERTIFFKD
DGNYKTRAEVKFEGDTLVNRIELKGIDFKEDGNILGHKLEYNYNSHNVYIMADKQKNGIKVNFKIRHNI
EDGSVQLADHYQQNTPIGDGPVLLPDNHYLSTQSKLSKDPNEKRDHMVLLEFVTAAGITLGMDELYKSG
LRSHHHHHHHH

ε_Pme_-UndB_GFP

ε_Pme_-UndB: _Pmen_4370_ gene with residues 158-189 replaced with residues 154-184 from _C427_4391_ gene (replaced residues highlighted in red)

SSG: Three residue long linker (Ser-Ser-Gly)

TEV: TEV-protease cleavage site

eGFP: enhanced Green fluorescent protein

His$_8$: 8xHis-tag

MEMSPSPASLNDQQRAAHIREQVMAHGNALRQRYPILQHQDALGAGILAFALCGMIGSAALYIGGHLPW
WACLLLNAFFASLTHELEHDLIHSMYFRKQPLPHNLMLALVWLARPSTINPWVRRHLHLNHHKVSGSE
ADMEERAITNGEPWGIARLLMV**CDGLLGLIIRSKQFSREIKGYNFFNVFNASL**PLGLLNWATWYLFLG
FHLLDWAAAALGAPIAWSASTLSVMQVVNVAVVVLVGPNVLRTFCLHFVSSNMHYYGDVEPGNVIQQT
QVLNPWWLWPLQAFCFNFGSSHAIHHFVVKEPFYIRQLTVPFAHRVMREMGVRFNDFGTFARANRWT
RRARTQQERASTASSGTENLYFQSGAAAAVSKGEELFTGVVPILVELDGDVNGHKFSVSGEGEGDATYGK
LTLKFICTTGKLPVPWPTLVTTLTYGVQCFSRYPDHMKQHDFFKSAMPEGYVQERTIFFKDDGNYKTRA
EVKFEGDTLVNRIELKGIDFKEDGNILGHKLEYNYNSHNVYIMADKQKNGIKVNFKIRHNIEDGSVQLAD
HYQQNTPIGDGPVLLPDNHYLSTQSKLSKDPNEKRDHMVLLEFVTAAGITLGMDELYKSGLRSHHHHH
HHH

ε_Pps_-UndB_GFP

ε_Pps_-UndB: _C427_4391_ gene with residues of 154-184 replaced with residues 158-189 from _Pmen_4370_ gene (replaced residues highlighted in red)

SSG: Three residue long linker (Ser-Ser-Gly)

TEV: TEV-protease cleavage site

eGFP: enhanced Green fluorescent protein

His_8: 8xHis-tag

MEMQNNQDKQDIKEIVAYIKNQERTLRSNHPFLAQQNALGLGLLLVSVCGFTAAGCLYFYAVIPAWCCIII
AALSASIAHEIEHDLIHQQYFKSNSAVYHFMMFMVWIIRPNTVNPWYRKGMHLNHHKTSGTPQDIEER
LVGNGIKSHTLRLLVV**GDNMMSSFIRWLRAKNPEHRRLILTRTLKVYA**PFVTVYYLIIYIFLLFHGVNFI
ADSSAVKLNTPVWLVSLMEWVNFAMVVWVAPNFLRSACLNFITSSMHYYGARFNLLEQTQVLNHWAF
MPFQWFCFNFGHTHSIHHFVPNQPFYIRQIISKQVNLLLKNKGVKFNDLVSIFAANHYKKIERSSSSGTEN
LYFQSGAAAAVSKGEELFTGVVPILVELDGDVNGHKFSVSGEGEGDATYGKLTLKFICTTGKLPVPWPTLV
TTLTYGVQCFSRYPDHMKQHDFFKSAMPEGYVQERTIFFKDDGNYKTRAEVKFEGDTLVNRIELKGIDF
KEDGNILGHKLEYNYNSHNVYIMADKQKNGIKVNFKIRHNIEDGSVQLADHYQQNTPIGDGPVLLPDNH
YLSTQSKLSKDPNEKRDHMVLLEFVTAAGITLGMDELYKSGLRSHHHHHHHH

ε*Nbr*-UndB_GFP

ε*Nbr*-UndB: *O3I_006310 gene from Nocardia brasiliensis* strain (ATCC700358) with residues 166-207 replaced with residues of 158-189 of *Pmen_4370* gene (replaced residues highlighted in red)

SSG: Three residue long linker (Ser-Ser-Gly)

TEV: TEV-protease cleavage site

eGFP: enhanced Green fluorescent protein

His_8: 8xHis-tag

MEMRLATRLPGERTLAPQDADRIAAIRGEIARVGDRWRVEHPWIAGHQNTIGAVIFLGAVLGVLGDAAL
YACGLLPWWATVLASAFWLSLLHEIEHDLIHAMYFRTNKWVHNAMLAGVWLLRPSTINPWVRRRLHL
HHHAVSGTESDLEERAISNGERWGGHRLLGL**GDNMMSSFIRWLRAKNPEHRRLILTRTLKVYA**PLSA
MHYGLWYLTVSAHVYELLGGTVGYPGAYRALDILAVTLLAPNAIRTFCLYFVSSNLHYYGDVEPHNVLQQ
TQVWTARWLWPVHALCFNFGGTHAIHHFVVRDPFYIREAIRAECQTILREHGVRFNDFGTFRRANRFG
LAVPPGAVRPSSGTENLYFQSGAAAAVSKGEELFTGVVPILVELDGDVNGHKFSVSGEGEGDATYGKLTLK
FICTTGKLPVPWPTLVTTLTYGVQCFSRYPDHMKQHDFFKSAMPEGYVQERTIFFKDDGNYKTRAEVKF
EGDTLVNRIELKGIDFKEDGNILGHKLEYNYNSHNVYIMADKQKNGIKVNFKIRHNIEDGSVQLADHYQ
QNTPIGDGPVLLPDNHYLSTQSKLSKDPNEKRDHMVLLEFVTAAGITLGMDELYKSGLRSHHHHHHHH

His_6-Fdx

His_6: 6xHis-tag

Fdx: Full-length Synpcc7942_0698 gene encoding ferredoxin from *Synechococcus elongatus* (strain ATCC 33912)

MGSSHHHHHHSQDMPSIRFIREDKEVFAADGANLRFKAVENQVDLYTFGGKMMNCGGYGQCGTCIVEI
VQGAENLSPRTSFEERKLKRKPDSYRLACQATVNGPVTVLTKPNPKEAQRETLIAQDLARPIPVTAPPAL
PQTETEVAGDPPSIATAET

His$_6$-FNR

His$_6$: 6xHis-tag

FNR: Full-length Ferredoxin--NADP reductase encoding synpcc7942_0978 gene from *Synechococcus elongatus* (strain ATCC 33912) (UniProt ID: Q31PL1)

MGSSHHHHHHSQDPMLNASVAGGAATTTYGNRLFIYEVIGLRQAEGEPSDSSIRRSGSTFFKVPYSRMN
QEMQRILRLGGKIVSIRPAEEAAANNGAAPLQAAAEEPAAAPTPAPAAKKHSAEDVPVNIYRPNKPFVG
KVLSNEPLVQEGGIGVVQHLTFDISEGDLRYIEGQSIGIIPDGTDDKGKPHKLRLYSIASTRHGDHVDDKTV
SLCVRQLQYQNEAGETINGVCSTFLCGLKPGDDVKITGPVGKEMLLPADTDANVIMMGTGTGIAPFRAYL
WRMFKDNERAINSEYQFNGKAWLIFGIPTTANILYKEELEALQAQYPDNFRLTYAISREQKNEAGGRMYI
QDRVAEHADEIWNLLKDEKTHVYICGLRGMEDGIDQAMTVAAAKEDVVWSDYQRTLKKAGRWHVET
Y

Supporting Methods

Recombinant expression and isolation of cellular membrane envelope fractions (CEF) of UndB

E. coli Rosetta (DE3) cells were transformed with the plasmids harboring *Pme-UndB*, *Pps-UndB*, and ε*Pme-UndB* genes, and *E. coli* (BL21DE3) cells were transformed with plasmid harboring *KatE-UndB* gene and grown on lysogeny broth (LB)-agar plate containing 100 µg/mL ampicillin. A 10 mL of primary culture in LB medium containing the same antibiotic was inoculated with a single colony from the corresponding plates and grown at 37 °C at 220 rpm overnight. A 5 mL primary culture was transferred to 500 mL terrific broth (TB) with 100 µg/mL ampicillin and grown at 37 °C at 220 rpm until the OD_{600} reached 0.6. The cultures were then cooled on ice for 10 min and induced with 0.5 mM isopropyl β- d-1-thiogalactopyranoside (IPTG) and 0.2 mM of $(NH_4)_2Fe(SO_4)_2.6H_2O$. The cells expressing KatE-UndB were supplemented with 0.1 mM 5-aminolevulinic acid for proper heme incorporation into the protein. The cultures were then transferred to an incubator shaker at 16 °C and grown at 220 rpm for 20 h.

For purification of *Pme*-UndB, KatE-UndB, and ε*Pme*-UndB, the cultures were centrifuged at 4,000 *g* for 45 min at 4 °C. The harvested cells of *Pme*-UndB and KatE-UndB from 1 L of culture (~10-12 g) were resuspended in 200 mL of buffer A (50 mM HEPES (4-(2-hydroxyethyl)-1-piperazineethanesulfonic acid), pH 8.0, 200 mM NaCl) supplemented with 0.5 mM PMSF (phenylmethylsulfonyl fluoride), and 50 µg/mL DNaseI. The cells expressing ε*Pme*-UndB were resuspended in a buffer B ((50 mM HEPES (4-(2-hydroxyethyl)-1-piperazineethanesulfonic acid), pH 7.0, 200 mM NaCl) supplemented with 0.5 mM PMSF, and 50 µg/mL DNaseI. The resuspended cells were lysed by sonication (45% amplitude, 2 sec on – 3 sec off, 3 cycles of 2 min each) using a sonicator (Q500, Qsonica USA). Cells were kept on ice during the sonication and rested for 5 min between sonication cycles. The cell lysates were centrifuged at 12,000 *g* for 10 min at 4 c to pellet the cell debris. The supernatant was collected and centrifuged at 150,000 *g* for 65 min at 4 °C using the Ti70 rotor in the optima XPN-100 ultracentrifuge (Beckman Coulter, USA) to isolate the CEF fractions. The isolated CEF fractions of *Pme*-UndB and KatE-UndB were homogenized in 10 mL buffer A, and that of ε*Pme*-UndB was homogenized in buffer B, respectively, supplemented with 0.5 mM PMSF and used for activity assays. The concentrations of the UndB in CEF is calculated based on fluorescent intensities of eGFP as described previously[1-2]. Specifically, we measured the eGFP fluorescence emission at 512 nm after excitation at 485 nm of the CEF containing UndB-eGFP. The protein concentration in CEF was measured based on the slope

obtained from the standard curve prepared by measuring the fluorescence intensities of purified UndB-eGFP protein in the same buffer. To account for the dampening of eGFP fluorescence in CEF samples, the fluoresce intensities were multiplied by 1.3, and the background was subtracted by measuring the fluorescence intensity of CEF of the cells expressing an empty vector, as per the previously published protocol(2).

Expression and purification of *Sel*-ferredoxin *Sel*-ferredoxin reductase from *Synechococcus elongatus* PCC7942

To express *Sel*-ferredoxin (*Sel*-Fdx) and *Sel*-ferredoxin reductase (*Sel*-FNR), BL21(DE3) *E. coli* cells were transformed with the pET-28b(+) plasmids harboring the genes encoding *Sel*-Fdx and *Sel*-FNR and grown overnight on the LB agar plate containing kanamycin (50 µg/mL) at 37 °C. A single colony was picked from the plates and grown overnight in a 10 mL LB media with the same antibiotic at 37 °C at 220 rpm. A 10 mL primary culture was inoculated into 1 L of TB media containing kanamycin (50 µg/mL) and grown at 37 °C at 220 rpm till the OD_{600} reached 0.6. To induce expression of *Sel*-Fdx, the culture was cooled down to 18 °C and supplemented with 0.5 mM IPTG and 0.2 mM of $(NH_4)_2Fe(SO_4)_2.6H_2O$. The culture was grown for 22 h at 18 °C before harvesting the cells by centrifuging the culture at 4,000 *g* for 40 min at 4 °C. The expression of *Sel*-FNR was induced with 0.5 mM IPTG after cooling down the culture to 25 °C. The culture was supplemented with 50 µM FAD and grown for 22 h at 25 °C at 220 rpm before harvesting by centrifugation at 4,000 *g* for 40 min at 4 °C.

For purification of *Sel*-Fdx, the cell pellet of wet weight ~5 g was resuspended in 50 mL of lysis buffer (50 mM Tris-HCl, pH 8.0, 250 mM NaCl, 10 mM imidazole, and 10% glycerol) supplemented with 5 mM β-mercaptoethanol (β-ME), 1 mM PMSF and 5 µg/mL DNaseI. The resuspended cells were subjected to sonication (50% amplitude, 2 sec ON – 3 sec OFF, 2 cycles of 2 min) using a sonicator (Q500, Qsonica USA). The cells were kept on ice during the sonication and were given a 5 min break between cycles. The cell debris was separated through centrifugation at 10,000 *g* for 45 min at 4 °C. After lysis, the supernatant containing soluble contents was incubated with 1 mL of Ni-NTA resin, pre-equilibrated with the lysis buffer for 1 h at 4°C. The slurry was transferred to a column, and the resin was washed with 10 column volumes (CV) of the lysis buffer. The resin was further washed with 20 CV of wash buffer (50 mM Tris-HCl, pH 8.0, 250 mM NaCl, 40 mM imidazole, and 10% glycerol) and eluted with elution buffer (50 mM Tris-HCl, pH 8.0, 250 mM NaCl, 300 mM imidazole, and 10% glycerol). The eluted fractions having brown-colored protein were pooled and desalted immediately in the desalting buffer (20 mM Tris-HCl, pH 8, 100 mM NaCl, and 10% glycerol) using a 5 mL desalting column (Bio-Scale Mini Bio-Gel P-6 cartridges, BioRad, USA, catalog no. 7324502). The active concentration of *Sel*-Fdx was calculated from its absorbance at 421 nm ($\mathcal{E}_{421}$ = 9690 M^{-1} cm^{-1})[3].

For purification of the *Sel*-FNR, the cell pellet was resuspended in the lysis buffer (50 mM sodium phosphate, pH 7.4, 250 mM NaCl, 10 mM imidazole, and 10% glycerol) supplemented with 1 mM PMSF and 5 µg/mL DNaseI. Cells were lysed by sonication (50% amplitude, 2 sec ON – 4 sec OFF, 3 cycles of 2 min with 5 min break in between) using a sonicator while keeping the cells on ice throughout the sonication. The supernatant containing soluble protein, after separation of cell debris by centrifugation, was incubated with 1 mL of Ni-NTA resin (pre-equilibrated with 10 CV of the lysis buffer) for 2 h at 4°C. The slurry was transferred to a column and washed with 20 CV of the wash buffer A (50 mM sodium phosphate, pH 7.4, 250 mM NaCl, 20 mM imidazole, and 10% glycerol), followed by 20 CV of the wash buffer B (50 mM sodium phosphate, pH 7.4, 250 mM NaCl, 45 mM imidazole and 10% glycerol). The elution of protein was carried out with 3 CV of the elution buffer (50 mM sodium phosphate, pH 7.4, 250 mM NaCl, 300 mM imidazole, and 10% glycerol). The yellow-colored fractions of eluted protein were pooled and desalted immediately

with the desalting buffer (50 mM potassium phosphate, pH 7.0, and 10% glycerol) using a 5 ml desalting column. The purity of the protein was analyzed by SDS-PAGE. The active concentration of *Sel*-FNR was measured from its absorbance at 456 nm ($\mathcal{E}_{456}$ = 10400 M^{-1} cm^{-1})[4].

Activity assays with regeneration system

Reactions were performed in 1.5 mL screw-capped glass vials with a reaction volume of 0.5 mL. The reaction mixture consisted of 1 µM UndB (Entries I and II, Fig. S1) or 0.1 µM KatE-UndB (Entries III and IV, Fig. S1) as present within CEF (isolated following the method described above). The assays were performed in assay buffer (50 mM HEPES, pH 8.0, 200 mM NaCl, 0.005 % (w/v) LMNG, 100 µM Fe $(NH_4)_2Fe(SO_4)_2{\cdot}6H_2O$, and 1 mM $CaCl_2$) and contained 1 mM lauric acid, 5 % (v/v) tergitol NP-10 as a cosolvent, 2.5 µM ferredoxin reductase, and 15 µM ferredoxin (Entries I and III, Fig. S1). To test the effect of the regeneration system, the reaction mixture of entries II and IV (Fig. S1) had 10 U/mL glucose dehydrogenase and 2 mM D-glucose. We note that we used 0.4 µM UndB and 1 mM substrate while doing experiments for the yield calculation. The reaction was initiated by the addition of 1 mM of NADPH for entries I and III and 100 µM NADPH for entries II and IV and performed at 25 °C for 1 h. The products were extracted in ethyl acetate and analyzed by GC-MS.

Activity assays with growing cells

For measuring the decarboxylation activity of UndB (*Pme*_UndB, *Pps*_UndB*, Pfl_pf-5*_UndB*, and Aba_AYE*_UndB) with growing cells, a small-scale secondary culture was started with 50 mL TB medium in a similar way as mentioned above. Once the OD_{600} reached 0.6, the culture was cooled on ice and induced with 0.5 mM IPTG and supplemented with 0.2 mM $(NH_4)_2Fe(SO_4)_2.6H_2O$ and shaken well. 475 µL of this culture was then aliquoted into a 2 mL glass vial containing 25 µL of substrate (lauric and palmitic of 10 mM stock concentrations prepared in ethanol). The vials were screw-capped, and the culture was left to grow for 18 h at 25 °C, 220 rpm. The alkene produced was then isolated by extracting the contents of the culture in 500 µL of ethyl acetate with internal standard (1-nonene). The organic layer was separated using centrifugation at 10,000 g for 10 min and analyzed by GC-MS.

Gas chromatography-mass spectrometry (GC-MS) analysis

The alkenes formed in the reactions were detected and quantified using GC-MS system (Agilent, 8890 GC coupled with the 5977B inert plus MSD) equipped with an HP-5MS UI column (Restek, 30 m x 0.25 mm x 0.25 µm, catalog no. 19091S-433UI). The helium carrier gas was maintained at a flow rate of 1.5 mL/min. A 5 µL of the sample was injected into the system using the autosampler (Agilent, ALS-G4513A) in the split mode with a split ratio of 5:1 with an inlet temperature of 250 °C and a total flow of 7.8 mL/min. The interface temperature was set to 250 °C. The oven temperature was held at 50 °C for 4 min, increased to 180 °C at 25 °C/min, then increased to 240 °C at 5 °C/min, and held for 5 min, and finally increased to 300 °C at 25 °C/min. The mass range for acquiring the data was 30-550 m/z, with the rest of the mass spectrometer parameters tuned automatically. Alkene signals were identified and quantified using calibration with authentic standards (TCI Chemicals, Japan) using the in-built analysis software (OpenLab CDS, Agilent, version 2.6).

Protein sequences of UndB homologs used for structural comparison

Class I

Pme*-UndB from *Pseudomonas mendocina strain ymp

MSPSPASLNDQQRAAHIREQVMAHGNALRQRYPILQHQDALGAGILAFALCGMIGSAALYIGGHLPWWA
CLLLNAFFASLTHELEHDLIHSMYFRKQPLPHNLMLALVWLARPSTINPWVRRHLHLNHHKVSGSEAD
MEERAITNGEPWGIARLLMVGDNMMSSFIRWLRAKNPEHRRLILTRTLKVYAPLGLLNWATWYLFLGF
HLLDWAAAALGAPIAWSASTLSVMQVVNVAVVVLVGPNVLRTFCLHFVSSNMHYYGDVEPGNVIQQTQ
VLNPWWLWPLQAFCFNFGSSHAIHHFVVKEPFYIRQLTVPFAHRVMREMGVRFNDFGTFARANRWTR
RARTQQERASTA

Pfl*I-UndB from *Pseudomonas fluorescens strain Pf0-1

MDRTSASPQRHNAAQRSAHIREVVLAKGVELRERYPILNHQDALGAGILVFALAGMIGSAALYVTGHMA
WWACLLLNAFFASLTHELEHDLIHSMYFRKQRVPHNLMMGLVWLARPSTINPWIRRHLHLNHHKVSG
TETDMEERAITNGEPWGFARLLMVGDNVMSAFIRMLRAKTWAHKFSIIKRTLKVYAPLALVHWGAWY
VFLGFHAANGIAYLMGSPIEWSATTLSVMQVIDIAAVVIIGPNVLRTFCLHFISSNMHYYGDVEPGNVLQQ
CQVLNPWWLWPLQAFCFNFGSSHGIHHFVVKEPFYIRQLTVPVAHKVMREMGVRFNDFGTFGRANRF
VRKENEGLAGKAIEVN

Pfl*II-UndB from *Pseudomonas fluorescens strain Pf-5

MQGISASPERMNAQQRAAHVRQVVLARGDELRRRFPLLRHQDALGAGILAFALSGMLGSALLYVTGHLA
WWACLLLNAFFASLTHELEHDLIHSMYFRKQRLPHNLMLGLVWLARPSTINPWVRRHLHLNHHKVSG
SESDIEERAITNGEPWGIARLLMVGDNMMAAFIRLLRAPGARRKLGILVRTLAVYAPLALLHWGAWYVF
LGFHGANGVAALLGSPIQWSQDTASLMHYVDIAVVVIIGPNVLRTFCLHFVSSNMHYYGDIEPGNVIQQT
QVLNPWWMWPLQAFCCNFGSTHGIHHFVVREPFYIRQMTASVAHKVMAEMGVRFNDFGTFARANRF
TRQEREAMQPAHNARA

Aba*-UndB from *Acinetobacter baumannii strain AYE

MTYIYKNPAGMTDSEKTEHIKKVVTAEGVALRKRHPILNHQNAIGAMILFISLVGMIATAVLYINHQLSA
WFAIPIIAFFASLTHELEHDLIHWMYFRKKPWAHHLMMGLVWLARPSTINPWKRRELHFNHHKNSGT
EVDLEERALTNGEQWSIRRLIAIGDNGLAVLFRIISASNWTVRKVIFKRAFMAYFPLGIIHWSLWYIFLGF
HAVDAVLSWANAPIAWSATTLNIMHVVNILTVVWVAPNVLRTFCLHFVTSNMHYYGDVELGNVIQQTQ
VLKPWWMMPFQLFCFNFGSTHAIHHFVVKEPFYIRQMTAPVAHKVMRDMGVRFNDVGTFKRANRW
NINDLSESKS

Pbr*-UndB from *Pseudomonas brassicacearum strain DF41

MHGTCASPERLNAQQRSAHIRQVVLARGEELRQRYPILRYQDALGAGILAFALVGMIGSALLYLNGHLAG
WACLLLNAFFASLTHELEHDLIHSMYFRKQRLPHNLMMGLVWLARPSTINPWIRRHLHLNHHKVSGSE
ADMEERAITNGEPWGLARLLMVGDNVMSAFIRLLRAKTWAHKRSILKRTLKVYFPLALLHWGAWYAF
LGFHGANGVASLLGTSVEWSATTLSVMHVIDIAAVVIIGPNVLRTFCLHFISSNMHYYGDIEPGNVIQQTQ
VLNPWWLWPLQAFCFNFGSSHGIHHFVVKEPFYIRQLTVPVAHKVMREMGVRFNDFGTFARANRFVR
QEGVVREAGGTVRV

Aol*-UndB from *Acinetobacter oleivorans strain DR1

MTYIYKNPAGMSDSEKTEHIKKVVTAEGVALRKRYPILNHQNAIGAMILFVSLVGMIATAVLYINHQLSA
WFAIPIIAFFASLTHELEHDLIHWMYFRKKPWAHHLMMGLVWLARPSTINPWKRRELHFNHHKNSGT
EVDLEERALTNGEQWSIRRLVAIGDNGFAVLLRIIAASNWTVRKVIFKRAFLAYFPLGIIHWSLWYIFLGF
HAVDMVASLANAPIAWSATTLNVMHVINVLTVVWIAPNVLRTFCLHFVTSNMHYYGDVELGNVIQQTQ

VLTPWWMMPFQLFCFNFGSTHAIHHFVVKEPFYIRQMTAPIAHKVMRDMGVRFNDVGTFKRANRWN
VNNLSESNT

Tpa*-UndB from *Turneriella parva strain H.DSM

MPTKIPPLSLTAADRVNRISRSIRMADRYLRRHFTFLNHQNLIGFSIWLGSIAGMIGMAALYYFDMAPAW
SVILVNAILASFLHELEHDLIHSLYFKETWIEKLMMWGVWAFRLNTPSPFYRKKIHLLHHKESGQFSDIE
EQMIGNGMKWGIKRIITMLDQGLAFLINARRVGKTAPRLDMKEMARAAFPFTYLYQGTSLVFLFGNAYL
LAMPHVDPAFVANADFVQLMALVNFMAVVIGLPNFIRQGALQIVSSSMHYFGDVNPDASVGLLEQCQVM
TTRSWYMLPFQLFCFNFGSTHGIHHFIVNQPFYLRQIAAGYSHAAMKKYGVRFDDHGSFARANRYGSTA
LTTGHSSLRSPSPLGEGVGG

Class II

Pps*-UndB from *Paraglaciecola psychrophile strain 170

MQNNQDKQDIKEIVAYIKNQERTLRSNHPFLAQQNALGLGLLLVSVCGFTAAGCLYFYAVIPAWCCIIIAAL
SASIAHEIEHDLIHQQYFKSNSAVYHFMMFMVWIIRPNTVNPWYRKGMHLNHHKTSGTPQDIEERLVG
NGIKSHTLRLLVVCDGLLGLIIRSKQFSREIKGYNFFNVFNASLPFVTVYYLIIYIFLLFHGVNFIADSSAVKL
NTPVWLVSLMEWVNFAMVVWVAPNFLRSACLNFITSSMHYYGARFNLLEQTQVLNHWAFMPFQWFC
FNFGHTHSIHHFVPNQPFYIRQIISKQVNLLLKNKGVKFNDLVSIFAANHYKKIERSS

Bce*-UndB from *Burkholderia cepacian strain 383

MSQPARTAFRNDADKVAYVRREVNAASDAIRARFPLLDNQNLVGATVMAVSVSAMLAIAWLYARGAIA
WYVALPLAAFVTSLIHELEHDLIHLMYFKKTPWAYHLMMALCWLTRPGTINPWTRRRMHLHHHKVSG
GESDLEEFGITNGERWGVKRLLMIADGMLAVVLRPTAMRRKVKQYVAAQPVQDPSERLQLRVEQVSSY
MPVGHVYYVLWHAFIVYHVGLFALHAFGFPVTVPAVVERVMSVVDFLAVVWLGPNFVRSFCINFVSSN
MHYFGDIDSRNVIQQTQVLNPWWMLPFQLFCFNFGSTHAIHHFVVRDPFYIRQLTARTAHAALREVGV
RFNDVGTFARANRWSGYRPSRGTRQAQADA

Nbr*-UndB from *Nocardia brasiliensis strain ATCC700358

MRLATRLPGERTLAPQDADRIAAIRGEIARVGDRWRVEHPWIAGHQNTIGAVIFLGAVLGVLGDAALYAC
GLLPWWATVLASAFWLSLLHEIEHDLIHAMYFRTNKWVHNAMLAGVWLLRPSTINPWVRRRLHLHH
HAVSGTESDLEERAISNGERWGGHRLLGLLDSVLGYATRPFRMRGLVAAYVARVARDPAEARRLAITTPL
AYFPLSAMHYGLWYLTVSAHVYELLGGTVGYPGAYRALDILAVTLLAPNAIRTFCLYFVSSNLHYYGDVEP
HNVLQQTQVWTARWLWPVHALCFNFGGTHAIHHFVVRDPFYIREAIRAECQTILREHGVRFNDFGTFR
RANRFGLAVPPGAVRP

Lbo*-UndB from *Leptospira borgpetersenii strain sv. Hardjo-bovis

MNSQWKTRNKPYKVFSEKEKTRKIIQWIRFWDDRIRNRFPYLSKYQDQIGFGIMIGSASGMILFAVLYIT
NLIPFWFCIVLNTIFASFLHEIEHDLIHNLYYKGRVKVQNFMLWVVWLFRANTVNPWFRREIHLLHHKL
SGNKEDVEERMIGNGVPFGLKRVLIMIDGNLALILQGRKVAKDAYLRLGKIKVPRTVGLYRETFFLLWYS
FLSVNAFHILNVLFGNPISEPSFLETNRSVLNSAAVVYLIPNWIRQTSLQVVSSNMHYYGNVPNVYHQTQ
VLNSWLVFPFHLFCFNFGATHGIHHFVVNQPFYLRQWVAFYVLSAMKRYGIRFNDFRSMWKSNSESLL
EENKIDFSKMTNFPISNSK

Bps*-UndB from *Burkholderia pseudomallei strain 1026b

MNKTLRFKNDAEKVAHVRNEVNAASDALRAKYPLLDNQNLIGAAVMAICVATLIGSAYLYAIGAIAWYVA
LPIATLATSLIHELEHDLIHLMYFKKTPWAYHAMMTLCWLTRPGTINPWTRRRMHLHHHKVSGGESDL
EEYGITNGERWGLKRLLMLADGMLAVALRPLGMRRKVLQYVAAQPAQARGDRVRLRIEQLMSYMPIGH
VYYVLWHAFIAYHAGLFALHALGYHPAVPALVQQTMHVVDFLAVTWLGPNFVRSFCINFISSNMHYYGD

IDSRNVVQQTQVLNPWWLIPVQLFCFNFGSTHAIHHFVVRDPFYIRQLTAKRAHAAMRAVGVRFNDIGT
FRRANRWNETRAA

Bth-UndB from Burkholderia _thailandensis strain H0587_

MNKTLRFKNDAEKVAYVRNEVNAASDALRAKYPLLDNQNLIGATVMALCVATLLGSAYLYATGMIAWY
VALPIATLATSLIHELEHDLIHLMYFKKTPWAYHVMMTLCWLTRPGTINPWTRRRMHLHHHKVSGGES
DLEEYGITNGERWGLKRLLMLADGMLAVALRPLGMRRKVLQYVAAQPAQERGDRVRLRIEQLMSYMPI
GHVYYVLWHAFIAYHVGLLALHALGYQPDVPMLVQQAMHVVDFLAVTWLGPNFVRSFCINFISSNMHY
YGDIDSRNVIQQTQVLNPWWLIPVQLFCFNFGSTHAIHHFVVRDPFYIRQLTAKRAHAAMRAVGVRFND
IGTFRRANRWNETRAA

Bce-UndB from _Burkholderia cenocepacia strain HI2424_

MSQPARTTFRHDADRVAYVRREVNAASDAIRARFPLLDNQNLVGATVMAVSVSAMLAIAWLYARGAIA
WYVALPLAAFITSLIHELEHDLIHLMYFKKTPWAYHLMMALCWLTRPGTINPWTRRRMHLHHHKVSG
GESDLEEFGITNGERWGVKRLLMIADGMLAVVLRPAAMRRKVKQYVAAQPVQDPSERLQLRIEQLSSYM
PIGHLYYTLWHAFIVYHVGLFALHALDVAVTVPAVVERVMSVVDFLAVVWLGPNFVRSFCINFVSSNMH
YFGDIDSRNVIQQTQVLNPWWMLPFQLFCFNFGSTHAIHHFVVRDPFYIRQLTARRAHAALREVGVRFN
DVGTFARANRWGGYRPSRRTHHAQADA

Computational Details

I. System set-up

In this study, we simulated both *Pme*-UndB and ε*Pme*-UndB, using lauric acid and palmitic acid as substrates, respectively. The initial atomic models of the proteins (*Pme*-UndB and ε*Pme*-UndB) were generated using the AlphaFold3 server[5] with default settings. Two Fe^{3+} ions were included as inputs during the AlphaFold3 computations. As a control, we also tested AlphaFold2[6] in conjunction with the JackHMMER algorithm for sequence comparison, which produced a similar structural prediction as AlphaFold3. In the AlphaFold3 model, the two iron centers are positioned approximately 5.9 Å apart (Fig. S5). Fe1 is coordinated by five histidine residues, while Fe2 is coordinated by four histidine residues.

For docking the lauric and palmitic acid substrates in the *Pme*-UndB and *εPme*-UndB protein models, we first determined the AlphaFold3 structure of *Pps*-UndB (specific to C16) with palmitic acid as the substrate. AlphaFold3 offers palmitic acid as a ligand option, and we observed the substrate docking in close proximity to the Fe2 atom. Subsequently, we used the *Pps*-UndB model bound with palmitic acid as a template to model substrate binding in the *Pme*-UndB and ε*Pme*-UndB models.

In the next step, we protonated both the protein and ligand. The protonation pattern of the protein was predicted using the H++ server[7] and further confirmed with the *propKa3* suite[8-9]. We oriented the protein for membrane insertion using the memembed code[10]. The ligand was protonated using the *reduce* tool from AmberTools23[11]. The entire protein-ligand system was then embedded in a lipid bilayer composed of POPE (1-palmitoyl-2-oleoyl-sn-glycero-3-phosphoethanolamine) and POPG (1-Palmitoyl-2-oleoyl-sn-glycero-3-(phospho-rac-(1-glycerol)) in a 4:1 ratio, with dimensions of 103 × 103 $Å^2$, using packmol-memgen[12]. This resulted in 280 POPE and 70 POPG lipid molecules distributed between the upper and lower leaflets. The lipid bilayer system was subsequently embedded in a water box with dimensions of 103 × 103 × 107.2 $Å^3$. To mimic physiological conditions, 0.20 M NaCl was added to the system. The final system contained 120,524 atoms for *Pme*-UndB and 112,683 atoms for ε*Pme*-UndB. In addition, we constructed two

additional setups: *Pme*-UndB with lauric acid and ε*Pme*-UndB with palmitic acid, using a 7:3 POPE:POPG membrane composition. In total, seven systems were constructed for molecular dynamics simulations (Table S1).

II. Parameterization

The classical force-field parameters for the di-iron cofactor and the ligands were derived using a systematic approach. For the di-iron assembly, the parametrization of both iron centers was carried out independently due to the large distance between them, which results in negligible orbital overlap. We employed a bonded model strategy to parametrize these metal-ligand complexes in this work. For the Fe1 center, a cluster model was extracted, consisting of His86, His90, His125, His129, His297, and Fe1 (Fig. S5*B*). A link hydrogen atom was placed at the Cβ position, and the geometry of the entire cluster model was fully optimized at the B3LYP/def2-TZVP level of theory with D4 dispersion correction[13]. During optimization, the Cβ and link hydrogen atoms were fixed in Cartesian space to avoid large-scale structural movements. A similar geometry optimization protocol was applied to the Fe2 center, where a cluster model consisting of Fe2 complexes with four histidine residues was used (His128, His258, His294, His298).

After optimization, the molecular Hessian was computed numerically at the B3LYP/def2-TZVP level of theory. The vibrational frequency data was then used to derive bonded parameters (bonds, angles, dihedrals, and impropers) using the Hess2FF code[14]. The charges of the entire cluster were calculated using the Merz–Kollman Restrained Electrostatic Potential (MK-RESP)[15-17] scheme, with the backbone atoms of histidine restrained to the charge of the link hydrogen atom, in line with the original AMBER force field implementation[18]. The MK-RESP charges were computed using the pyRESP[19] code, while the non-bonded parameters for Fe^{2+} were derived from available data[20-21] consistent with the TIP3P water model. All quantum chemical calculations were performed using the Turbomole 7.5 suite[22].

For the lauric and palmitic acid ligands, geometry optimization was first performed at the B3LYP/def2-TZVP level of theory. The MK-RESP charges were then computed at the HF/6-31G* level of theory, consistent with the charge derivation in the original AMBER force field. Bonded parameters for the ligands were assigned using the GAFF2 (General Amber Force Field) force field[23].

The Amber14SB force field[24] was used to describe the protein system, and the LIPID21[25] force field was employed for the lipid bilayers. The TIP3P[26] model was used for water molecules, and Na^{+} and Cl^{-} ions were described using the Joung–Cheatham parameters[27-28], consistent with the TIP3P water model.

III. Molecular dynamics simulations

All systems were first energy minimized to remove geometric clashes. For this, the system was optimized for 20,000 steps. During the equilibration phase, the temperature was systematically raised from 0 to 100 K over a short 5 ps propagation in the *NVT* ensemble. Subsequently, the temperature was further increased from 100 to 303 K during a 100 ps propagation in the *NPT* ensemble, with restraints (10 kcal/mol/Å^2) applied to the Cα atoms. Temperature control was maintained using Langevin dynamics[29] with a collision frequency of 5 ps^{-1}.

In the next step, the restraints were gradually reduced at a rate of 2 kcal mol^{-1} Å^{-2} per 200 ps, lowering them from 10 to 8 kcal mol^{-1} Å^{-2}. Following this, unrestrained MD simulations were conducted for 70 ns to allow the protein, lipid bilayer, and water to equilibrate further. The last snapshot from the equilibration phase was used as the starting point for the production simulations. Production simulations were performed in the *NPT* ensemble, with pressure controlled anisotropically using the Berendsen barostat[30] (relaxation time of 2 ps, at 1 bar). The SHAKE algorithm[31] was used to constrain bonds involving hydrogen atoms, with an integration time step of 2 fs. Electrostatic interactions were treated using the Particle Mesh Ewald (PME)[32] method with a cutoff of 10 Å. Frames were saved every 20 ps for data analysis. Details on the production simulations of various simulated systems are presented in Table S1.

Energy minimizations were performed using the CPU version of the *pmemd* engine[33-35], while the GPU version (*pmemd.cuda*) was used to propagate the equilibration and production simulations. All classical MD simulations were carried out using the AMBER24 suite[36-37], and visualization was performed using PyMOL (The PyMOL Molecular Graphics System, Version 1.2r3pre, Schrödinger, LLC) and Visual Molecular Dynamics (VMD)[38].

Supporting Figures and Tables

A

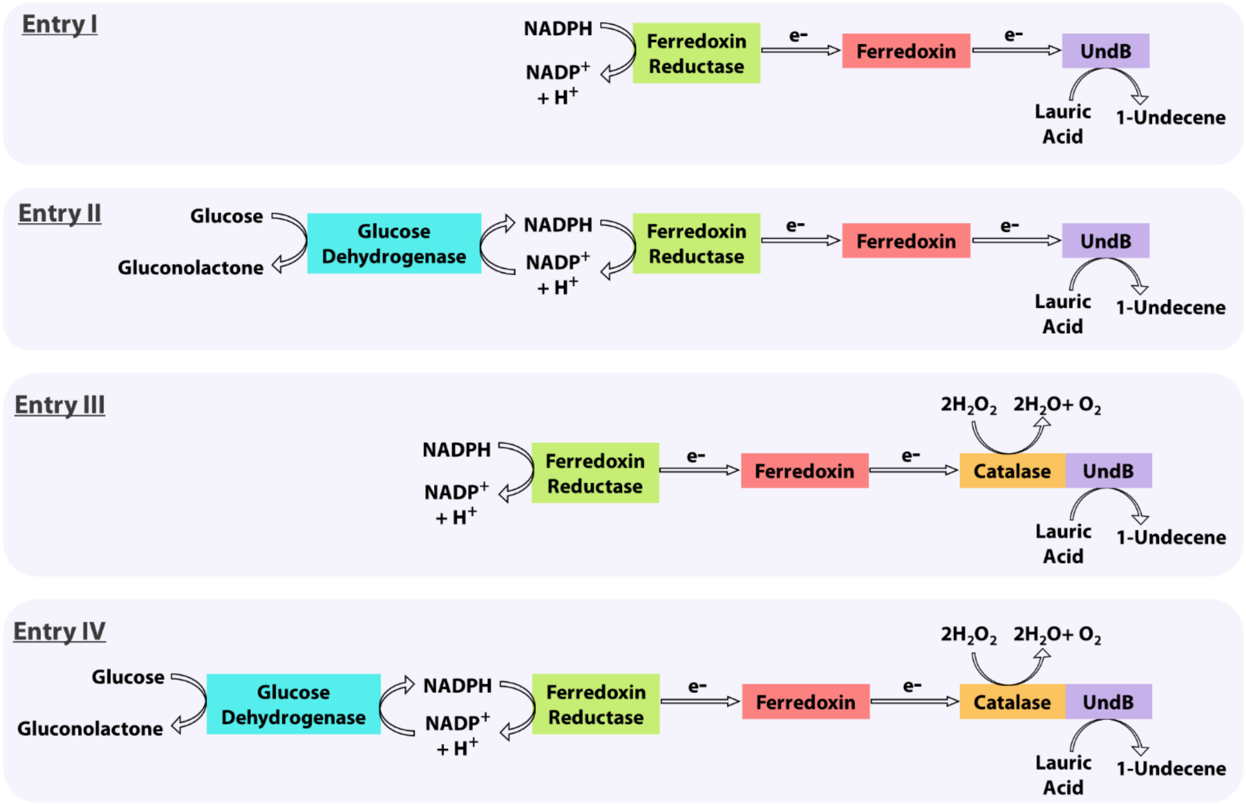

B

Entry	UndB	KatE-UndB	NADPH	NADPH regeneration system	TTN
I	+	-	+[a]	-	13
II	+	-	+[a]	+	23
III	-	+	+[b]	-	265
IV	-	+	+[b]	+	915

Figure S1. Production of 1-undecene from lauric acid using various UndB-based biocatalytic systems. (*A*) Schematics of the various biocatalytic systems utilized in this study. (*B*) Activities of various biocatalytic systems. Superscripts [a] and [b] denote the NADPH used at the concentration of 1 mM and 100 μM, respectively. Reactions were performed with 1 μM of UndB (Entries I and II) or 0.1 μM KatE-UndB (Entries III and IV), 2.5 μM ferredoxin reductase, 15 μM ferredoxin, 10 U/mL glucose dehydrogenase (Entries II and IV) and 2 mM D-glucose (Entries I and III) for 1 h at 25 °C as described above.

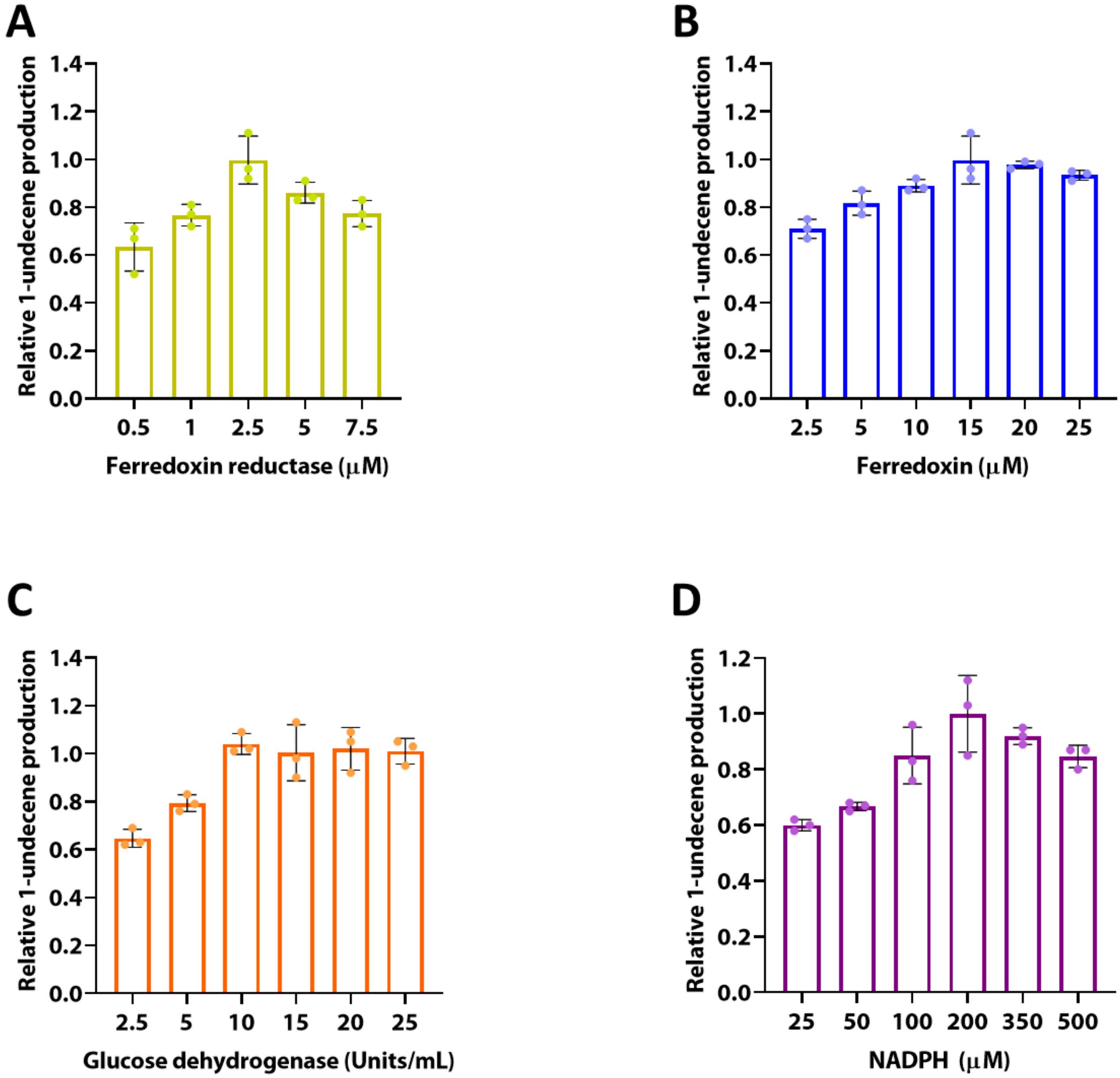

Figure S2. Optimization of various components of the cell-free biocatalytic system. Relative production of 1-undecene using varying concentrations of (*A*) ferredoxin reductase, (*B*) ferredoxin, (*C*) glucose dehydrogenase, and (*D*) NADPH. Reactions were performed with 0.1 μM of UndB, 1mg/mL of catalase, and 1 mM lauric acid for 6 h at 25 °C. Error bars represent the standard deviation (SD) of triplicate (n=3) data.

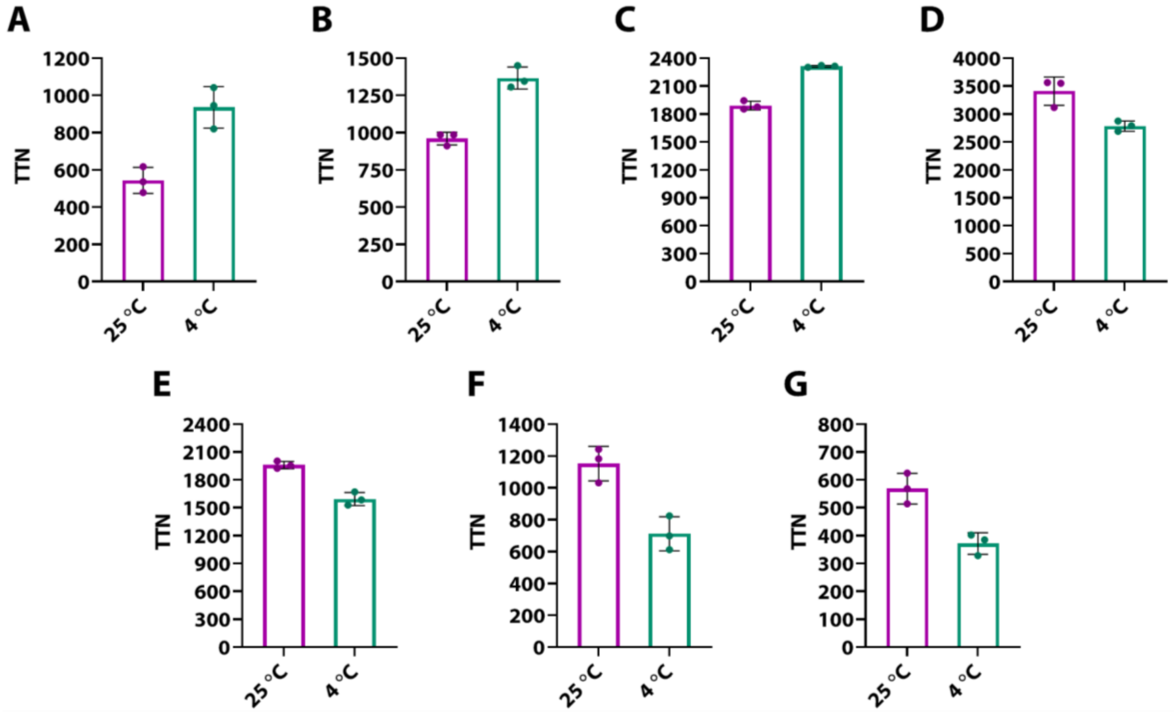

Figure S3. Activity of the cell-free biocatalytic system at different temperatures. Activity analysis of the cell-free biocatalytic system at 25 °C and 4 °C for the production of corresponding 1-alkenes from 1 mM of (*A*) hexanoic acid, (*B*) octanoic acid, (*C*) decanoic acid, (*D*) lauric acid, (*E*) myristic acid, (*F*) palmitic acid, and (*G*) stearic acid. Experiments were performed with 0.1 μM of UndB, 1 mg/mL of catalase, 2.5 μM ferredoxin reductase, 15 μM ferredoxin, 10 U/mL glucose dehydrogenase, 2 mM D-glucose, and 200 uM of NADPH for 6 h at 25 °C. as described in Methods, and error bars represent the standard deviation (SD) of triplicate (n=3) data.

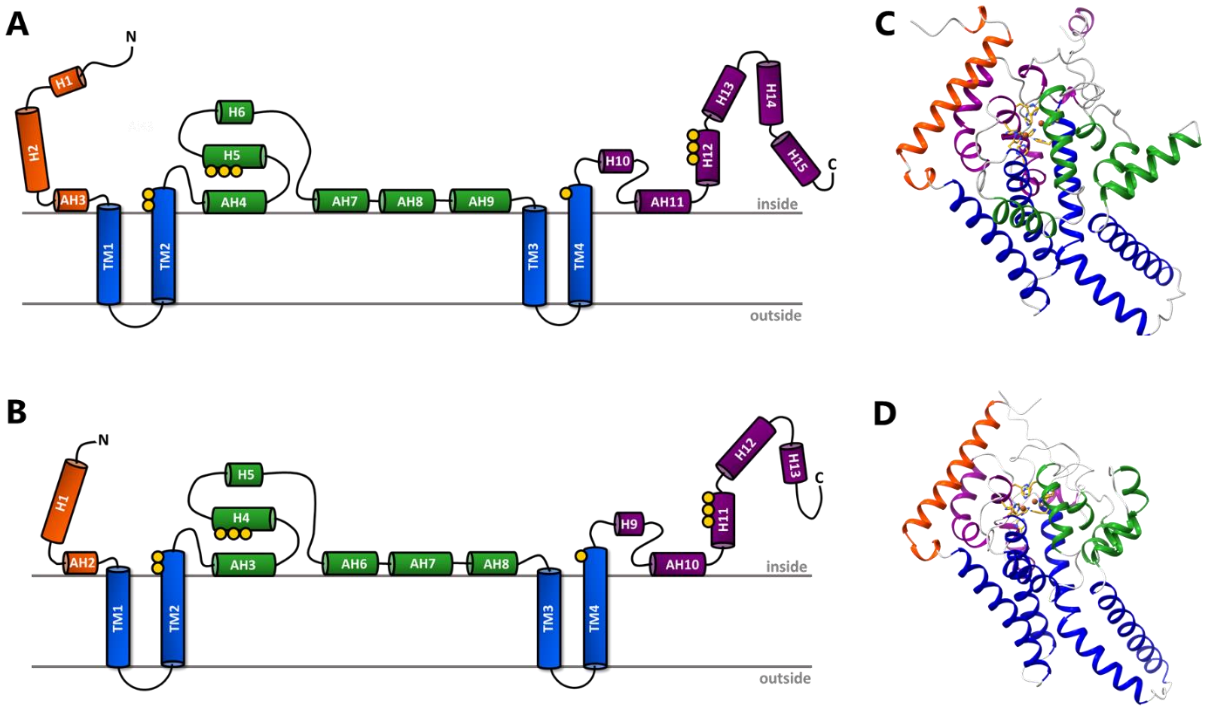

Figure S4. Topological representation of UndB homologs : The secondary structure topology of UndB homologs (A) *Pme*-UndB , (B) *Pps*-UndB - with a cylindrical representation of various soluble helices (Hx), amphipathic helices (AHx), and transmembrane helices (TMH1-TMH4). The AlphaFold3 predicted structure of UndB homologs (C) *Pme*-UndB, (D) *Pps*-UndB[39]. Helices are color-coded based on their presence within the structure. The cytosolic N-terminal helices are colored orange, the helices between the transmembrane helices (TMH2 and TMH3) are colored green, and the C-terminal helices are represented with a violet color. The presence of conserved histidine residues is shown with yellow-colored circles.

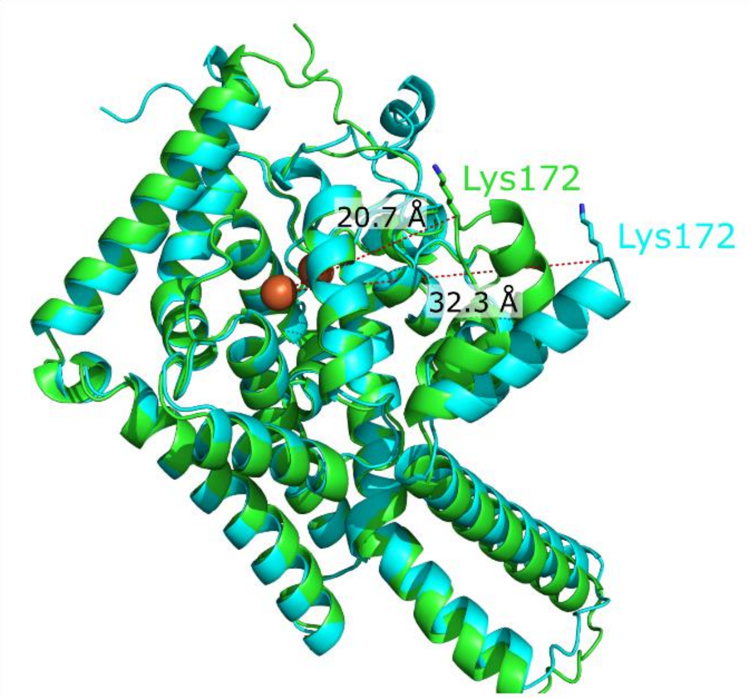

Figure S5. Representation of annotated distances between active site and the helix. The distance between diiron active site and the helix corresponding to residues 158-189 in *Pme*-UndB (cyan blue) and residues 154-184 in *Pps*-UndB (green) is 32.3 Å and 20.7 Å respectively.

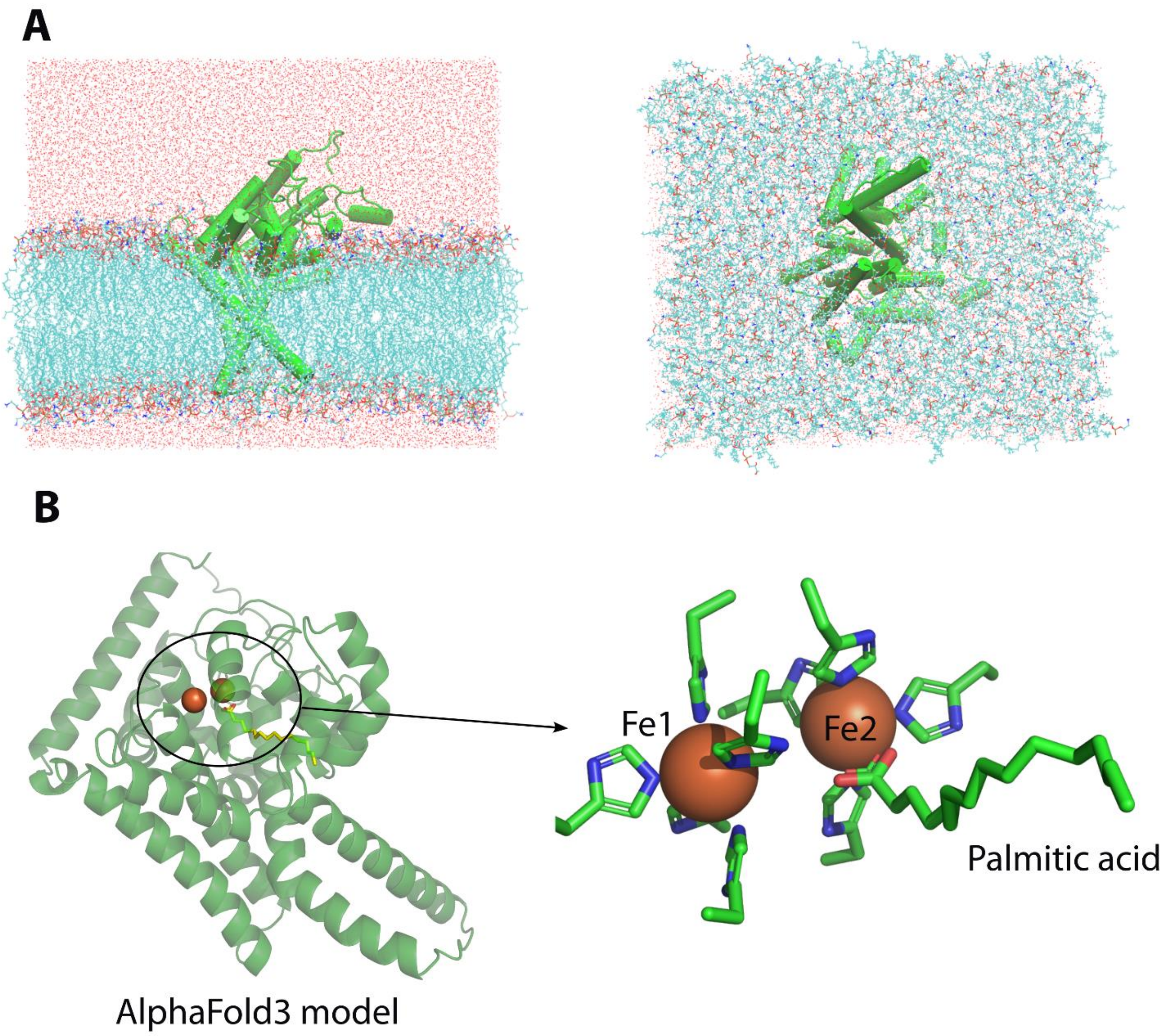

Figure S6. All-atom model of UndB-substrate. (*A*) Side (left) and top (right) views of the all-atom model of UndB embedded in a lipid bilayer and water box. (*B*) AlphaFold3-predicted three-dimensional structure of *Pps*-UndB bound to two Fe atoms and palmitic acid as the substrate. Fe1 and Fe2 are coordinated by five and four histidine residues, respectively. Palmitic acid is bound to the Fe2 atom in the AlphaFold3 model. The distance between the two iron atoms in the AlphaFold3 model is 5.9 Å.

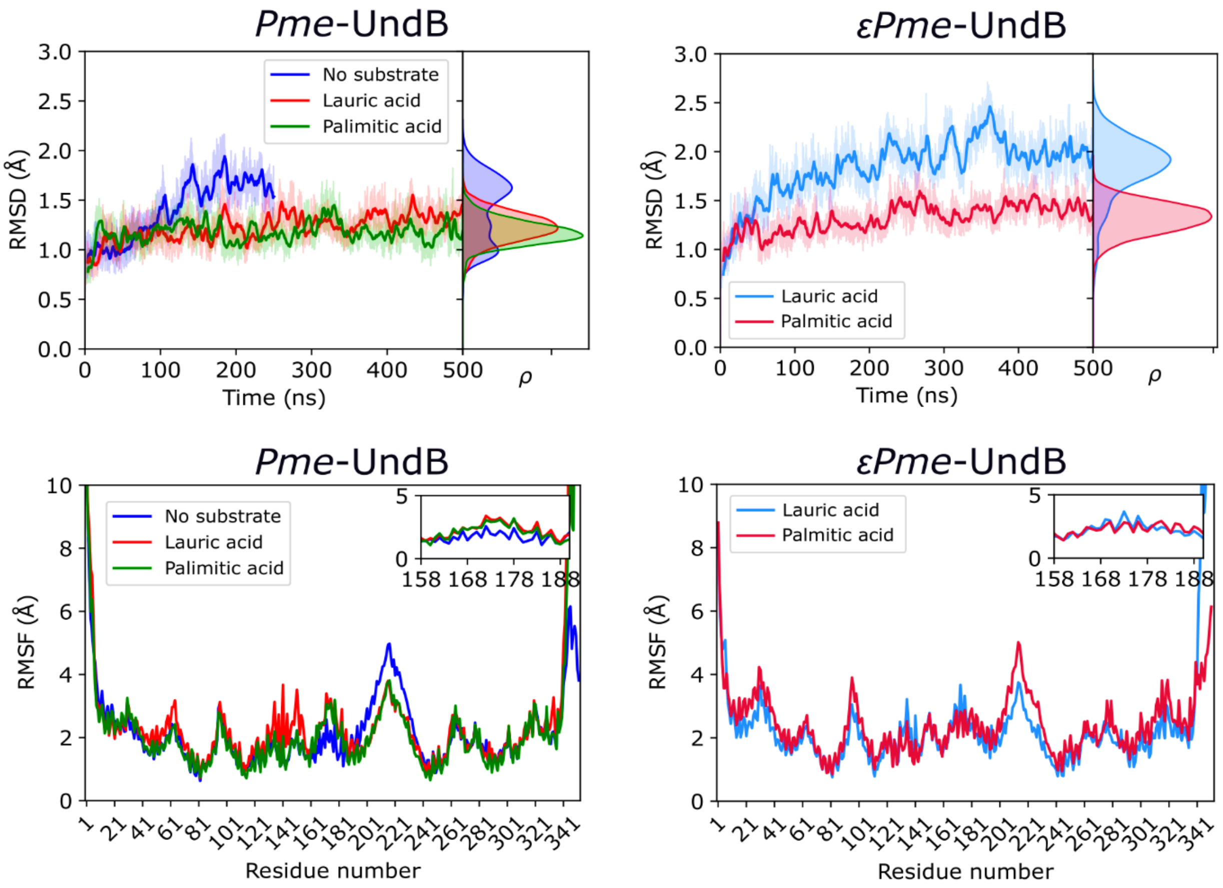

Figure S7. Molecular dynamics simulations of UndB-substrate systems. (Top) Root mean square deviation (RMSD) evolution of *Pme*-UndB (three systems: apo, lauric acid, and palmitic acid as substrates) and ε*Pme*-UndB (two systems: lauric acid and palmitic acid as substrates) along the molecular dynamics trajectories. (Bottom) Root mean square fluctuation (RMSF) of *Pme*-UndB (three systems: apo, lauric acid, and palmitic acid as substrates) and ε*Pme*-UndB (two systems: lauric acid and palmitic acid as substrates). (inset) RMSF of residue 158-189.

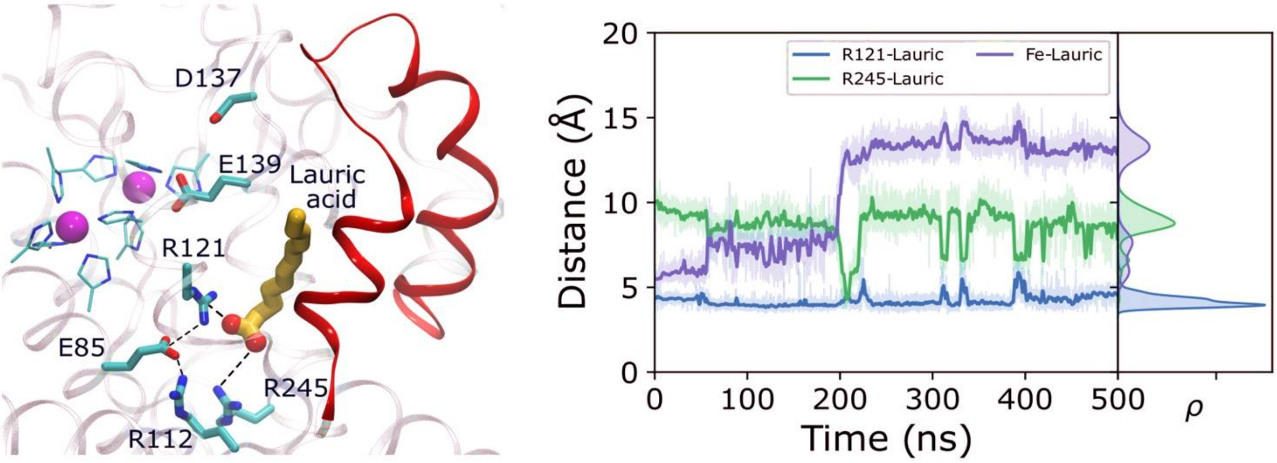

Figure S8. Dynamics of ε*Pme*-UndB-Lauric system. (*left*) Stable substrate-binding site for lauric acid in ε*Pme*-UndB. The secondary structure comprising residues 158-189 is highlighted in red. (*right*) Corresponding distance evolution between the diiron site, key residues (R121, Y188, and R245), and the substrate from MD simulations is shown.

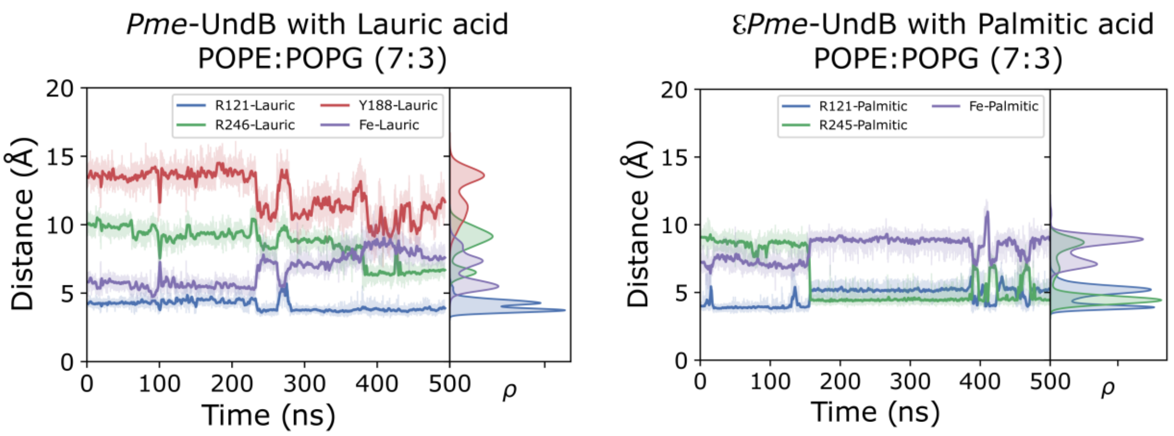

Figure S9. Dynamics of *Pme*-UndB and ε*Pme*-UndB with a 7:3 POPE:POPG membrane composition. Time evolution of distances between the diiron site, key residues (R121, Y188, and R245/R246), and lauric acid (*left*) or palmitic acid (*right*) obtained from MD simulations of *Pme*-UndB and ε*Pme*-UndB, respectively.

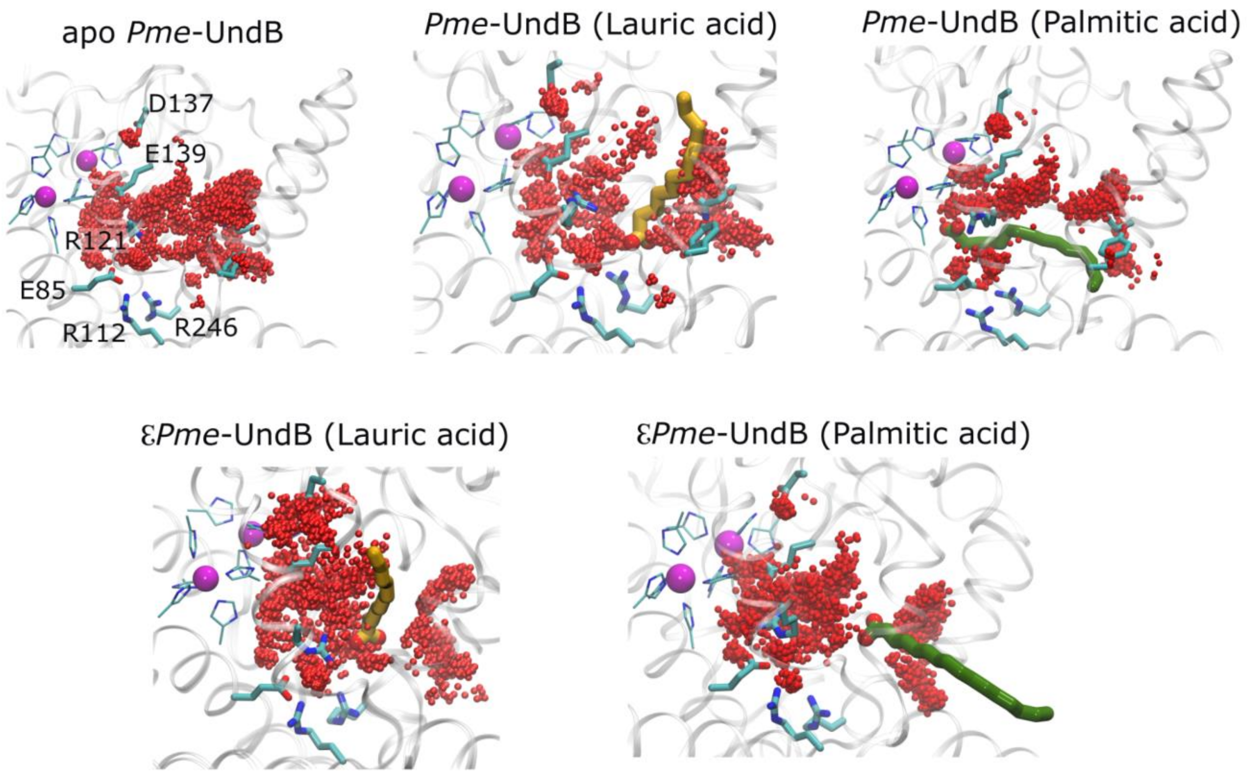

Figure S10. Hydration in UndB-substrate systems. Clustering of water molecules in the substrate cavity of *Pme*-UndB (three systems: apo, lauric acid, and palmitic acid as substrates) and ε*Pme*-UndB (two systems: lauric acid and palmitic acid as substrates), obtained from molecular dynamics simulations. Red spheres represent the oxygen atoms of the water molecules.

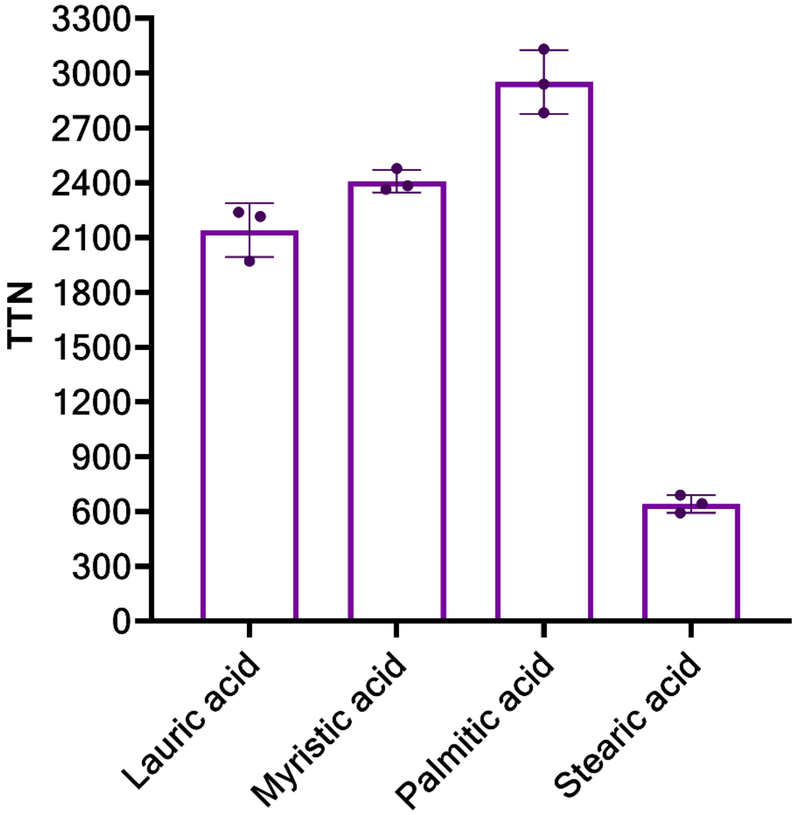

Figure S11. Activity of the cell-free biocatalytic system using the engineered ε*Pme*-UndB. Activity analysis of the cell-free biocatalytic system using the engineered ε*Pme*-UndB at 25 °C for the production of corresponding 1-alkenes from 1 mM of lauric acid, myristic acid, palmitic acid, and stearic acid, respectively. Experiments were performed with 0.1 μM UndB, 1 mg/mL catalase, 2.5 μM ferredoxin reductase, 15 μM ferredoxin, 10 U/mL glucose dehydrogenase, 2 mM D-glucose, and 200 μM NADPH as described in Methods, and error bars represent the standard deviation (SD) of triplicate (n=3) data.

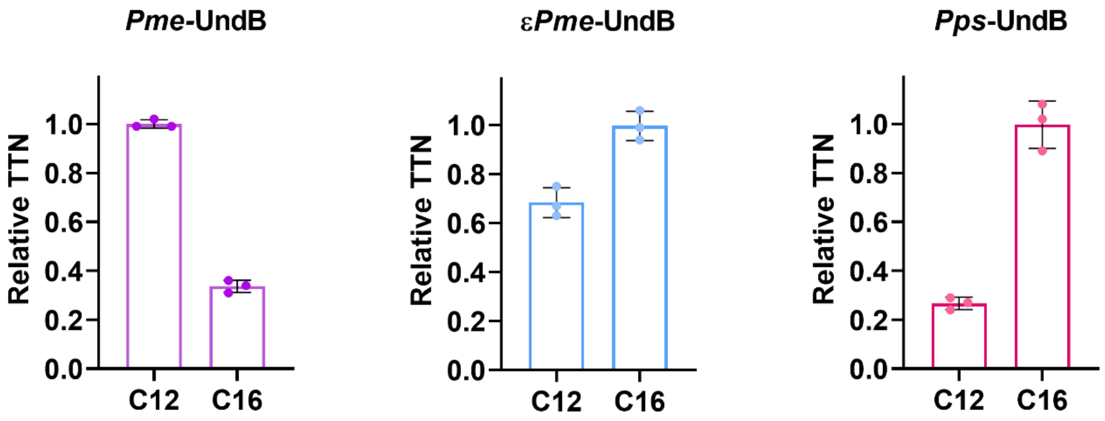

Figure S12. Relative activities of various UndB constructs. Relative 1-alkenes production from lauric acid (C12) and palmitic acid (C16) by whole cells expressing (C) *Pme*-UndB, ε*Pme*-UndB and *Pps*-UndB. Experiments were performed with growing cells as described in Methods. The error bars represent the standard deviation (SD) of triplicate (n=3) data.

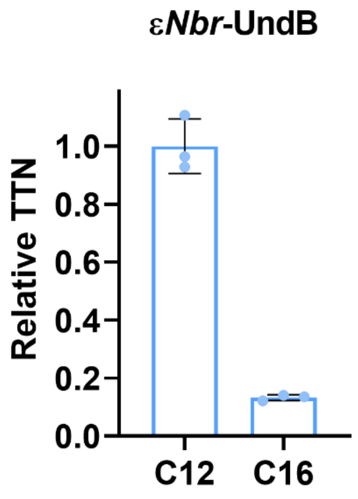

Figure S13: Relative activity of engineered *Nbr*-UndB construct (εNbr-UndB). Relative 1-alkenes production from lauric acid (C12) and palmitic acid (C16) by whole cells expressing ε*Nbr-UndB.* The error bars represent the standard deviation (SD) of triplicate (n=3) data.

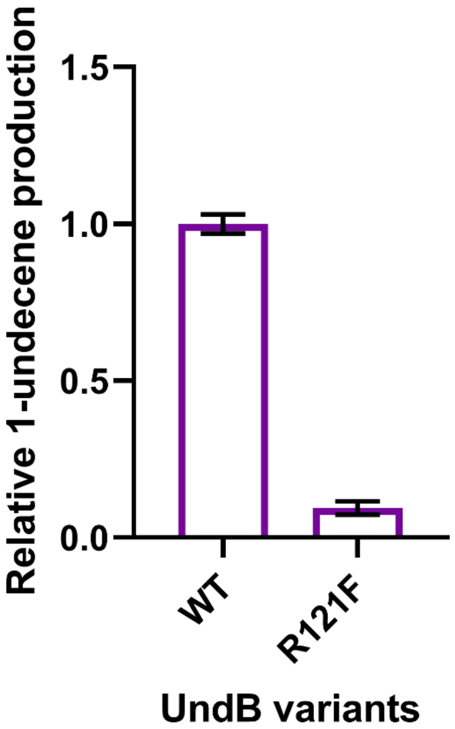

Figure S14. Relative activity of WT UndB and R121F mutant. Comparative activity analysis WT UndB and R121F mutant at 25 °C for the production of 1-undecene from lauric acid. Experiments were performed with 0.1 μM UndB as described in Methods, and error bars represent the standard deviation (SD) of triplicate (n=3) data.

Table S1. Details on the UndB-ligand systems propagated in all-atom molecular dynamics simulations.

Protein System	Membrane Composition	Ligand	Simulation time scale
Pme-UndB	POPE:POPG (4:1)	No Ligand	250 ns
Pme-UndB	POPE:POPG (4:1)	Lauric acid	500 ns
Pme-UndB	POPE:POPG (4:1)	Palmitic acid	500 ns
ε*Pme*-UndB	POPE:POPG (4:1)	Lauric acid	500 ns
ε*Pme*-UndB	POPE:POPG (4:1)	Palmitic acid	500 ns
Pme-UndB	POPE:POPG (7:3)	Lauric acid	500 ns
ε*Pme*-UndB	POPE:POPG (7:3)	Palmitic acid	500 ns
**Cumulative simulations time**			**3.25 µs**

Table S2. Sequence similarities and identities of UndB homologs (Class I and Class II).

Class	UndB Homologs	Sequence Identity	Sequence Similarity
I	*Pme*-UndB	*100 %*	*100 %*
I	*Pfl*I-UndB	*75 %*	*83 %*
I	*Pfl*II-UndB	*75 %*	*85 %*
I	*Aba*-UndB	*60 %*	*74 %*
I	*Pbr*-UndB	*77 %*	*85 %*
I	*Aol*-UndB	*61 %*	*74 %*
I	*Tpa*-UndB	*36 %*	*52 %*
II	*Pps*-UndB	*37 %*	*55 %*
II	*Bce*-UndB	*50 %*	*67 %*
II	*Nbr*-UndB	*47 %*	*61 %*
II	*Lbo*-UndB	*36 %*	*55 %*
II	*Bps*-UndB	*49 %*	*64 %*
II	*Bth*-UndB	*48 %*	*64 %*
II	*Bce*-UndB	*50 %*	*65 %*

Supporting Movies

Movie S1: Movie of the molecular dynamics of *Pme*-UndB bound to lauric acid. Lauric acid is depicted in yellow. Key residues in the central ion-pair network, as well as other residues interacting with the substrate, are also highlighted.

Movie S2: Movie of the molecular dynamics of *Pme*-UndB bound to palmitic acid. Palmitic acid is depicted in green. Key residues in the central ion-pair network, as well as other residues interacting with the substrate, are also highlighted.

Movie S3: Movie of the molecular dynamics of ε*Pme*-UndB bound to palmitic acid. Palmitic acid is depicted in green. Key residues in the central ion-pair network, as well as other residues interacting with the substrate, are also highlighted.

Movie S4: Movie of the molecular dynamics of ε*Pme*-UndB bound to lauric acid. Lauric acid is depicted in yellow. Key residues in the central ion-pair network, as well as other residues interacting with the substrate, are also highlighted.

SI References

1. Iqbal, T.; Murugan, S.; Das, D., A chimeric membrane enzyme and an engineered whole-cell biocatalyst for efficient 1-alkene production. *Science Advances* **2024,** *10* (26), eadl2492.
2. Drew, D.; Lerch, M.; Kunji, E.; Slotboom, D.-J.; de Gier, J.-W., Optimization of membrane protein overexpression and purification using GFP fusions. *Nature methods* **2006,** *3* (4), 303-313.
3. Zhang, W.; Du, L.; Li, F.; Zhang, X.; Qu, Z.; Han, L.; Li, Z.; Sun, J.; Qi, F.; Yao, Q.; Sun, Y.; Geng, C.; Li, S., Mechanistic Insights into Interactions between Bacterial Class I P450 Enzymes and Redox Partners. *ACS Catalysis* **2018,** *8* (11), 9992-10003.
4. Batie, C. J.; Kamin, H., The relation of pH and oxidation-reduction potential to the association state of the ferredoxin . ferredoxin:NADP+ reductase complex. *Journal of Biological Chemistry* **1981,** *256* (15), 7756-7763.
5. Abramson, J.; Adler, J.; Dunger, J.; Evans, R.; Green, T.; Pritzel, A.; Ronneberger, O.; Willmore, L.; Ballard, A. J.; Bambrick, J.; Bodenstein, S. W.; Evans, D. A.; Hung, C. C.; O'Neill, M.; Reiman, D.; Tunyasuvunakool, K.; Wu, Z.; Zemgulyte, A.; Arvaniti, E.; Beattie, C.; Bertolli, O.; Bridgland, A.; Cherepanov, A.; Congreve, M.; Cowen-Rivers, A. I.; Cowie, A.; Figurnov, M.; Fuchs, F. B.; Gladman, H.; Jain, R.; Khan, Y. A.; Low, C. M. R.; Perlin, K.; Potapenko, A.; Savy, P.; Singh, S.; Stecula, A.; Thillaisundaram, A.; Tong, C.; Yakneen, S.; Zhong, E. D.; Zielinski, M.; Zidek, A.; Bapst, V.; Kohli, P.; Jaderberg, M.; Hassabis, D.; Jumper, J. M., Accurate structure prediction of biomolecular interactions with AlphaFold 3. *Nature* **2024,** *630* (8016), 493-500.
6. Jumper, J.; Evans, R.; Pritzel, A.; Green, T.; Figurnov, M.; Ronneberger, O.; Tunyasuvunakool, K.; Bates, R.; Žídek, A.; Potapenko, A.; Bridgland, A.; Meyer, C.; Kohl, S. A. A.; Ballard, A. J.; Cowie, A.; Romera-Paredes, B.; Nikolov, S.; Jain, R.; Adler, J.; Back, T.; Petersen, S.; Reiman, D.; Clancy, E.; Zielinski, M.; Steinegger, M.; Pacholska, M.; Berghammer, T.; Bodenstein, S.; Silver, D.; Vinyals, O.; Senior, A. W.; Kavukcuoglu, K.; Kohli, P.; Hassabis, D., Highly accurate protein structure prediction with AlphaFold. *Nature* **2021,** *596* (7873), 583-589.
7. Gordon, J. C.; Myers, J. B.; Folta, T.; Shoja, V.; Heath, L. S.; Onufriev, A., H++: a server for estimating p Ka s and adding missing hydrogens to macromolecules. *Nucleic Acids Research* **2005,** *33* (suppl_2), W368-W371.
8. Søndergaard, C. R.; Olsson, M. H. M.; Rostkowski, M.; Jensen, J. H., Improved Treatment of Ligands and Coupling Effects in Empirical Calculation and Rationalization of pKa Values. *Journal of Chemical Theory and Computation* **2011,** *7* (7), 2284-2295.

9. Olsson, M. H. M.; Søndergaard, C. R.; Rostkowski, M.; Jensen, J. H., PROPKA3: Consistent Treatment of Internal and Surface Residues in Empirical pKa Predictions. *Journal of Chemical Theory and Computation* **2011,** *7* (2), 525-537.
10. Nugent, T.; Jones, D. T., Membrane protein orientation and refinement using a knowledge-based statistical potential. *BMC Bioinformatics* **2013,** *14*, 276.
11. Word, J. M.; Lovell, S. C.; Richardson, J. S.; Richardson, D. C., Asparagine and glutamine: using hydrogen atom contacts in the choice of side-chain amide orientation11Edited by J. Thornton. *Journal of Molecular Biology* **1999,** *285* (4), 1735-1747.
12. Schott-Verdugo, S.; Gohlke, H., PACKMOL-Memgen: A Simple-To-Use, Generalized Workflow for Membrane-Protein–Lipid-Bilayer System Building. *Journal of Chemical Information and Modeling* **2019,** *59* (6), 2522-2528.
13. Caldeweyher, E.; Ehlert, S.; Hansen, A.; Neugebauer, H.; Spicher, S.; Bannwarth, C.; Grimme, S., A generally applicable atomic-charge dependent London dispersion correction. *The Journal of Chemical Physics* **2019,** *150* (15), 154122.
14. Nilsson, K.; Lecerof, D.; Sigfridsson, E.; Ryde, U., An automatic method to generate force-field parameters for hetero-compounds. *Acta Crystallographica Section D* **2003,** *59* (2), 274-289.
15. Bayly, C. I.; Cieplak, P.; Cornell, W.; Kollman, P. A., A well-behaved electrostatic potential based method using charge restraints for deriving atomic charges: the RESP model. *The Journal of Physical Chemistry* **1993,** *97* (40), 10269-10280.
16. Duan, Y.; Wu, C.; Chowdhury, S.; Lee, M. C.; Xiong, G.; Zhang, W.; Yang, R.; Cieplak, P.; Luo, R.; Lee, T.; Caldwell, J.; Wang, J.; Kollman, P., A point-charge force field for molecular mechanics simulations of proteins based on condensed-phase quantum mechanical calculations. *Journal of Computational Chemistry* **2003,** *24* (16), 1999-2012.
17. Li, P.; Merz, K. M., Jr., Metal Ion Modeling Using Classical Mechanics. *Chemical Reviews* **2017,** *117* (3), 1564-1686.
18. Cornell, W. D.; Cieplak, P.; Bayly, C. I.; Gould, I. R.; Merz, K. M.; Ferguson, D. M.; Spellmeyer, D. C.; Fox, T.; Caldwell, J. W.; Kollman, P. A., A Second Generation Force Field for the Simulation of Proteins, Nucleic Acids, and Organic Molecules. *Journal of the American Chemical Society* **1995,** *117* (19), 5179-5197.
19. Zhao, S.; Wei, H.; Cieplak, P.; Duan, Y.; Luo, R., PyRESP: A Program for Electrostatic Parameterizations of Additive and Induced Dipole Polarizable Force Fields. *Journal of Chemical Theory and Computation* **2022,** *18* (6), 3654-3670.
20. Li, P.; Song, L. F.; Merz, K. M., Jr., Parameterization of Highly Charged Metal Ions Using the 12-6-4 LJ-Type Nonbonded Model in Explicit Water. *The Journal of Physical Chemistry B* **2015,** *119* (3), 883-895.
21. Li, P.; Roberts, B. P.; Chakravorty, D. K.; Merz, K. M., Jr., Rational Design of Particle Mesh Ewald Compatible Lennard-Jones Parameters for +2 Metal Cations in Explicit Solvent. *Journal of Chemical Theory and Computation* **2013,** *9* (6), 2733-2748.
22. Balasubramani, S. G.; Chen, G. P.; Coriani, S.; Diedenhofen, M.; Frank, M. S.; Franzke, Y. J.; Furche, F.; Grotjahn, R.; Harding, M. E.; Hättig, C.; Hellweg, A.; Helmich-Paris, B.; Holzer, C.; Huniar, U.; Kaupp, M.; Marefat Khah, A.; Karbalaei Khani, S.; Müller, T.; Mack, F.; Nguyen, B. D.; Parker, S. M.; Perlt, E.; Rappoport, D.; Reiter, K.; Roy, S.; Rückert, M.; Schmitz, G.; Sierka, M.; Tapavicza, E.; Tew, D. P.; van Wüllen, C.; Voora, V. K.; Weigend, F.; Wodyński, A.; Yu, J. M., TURBOMOLE: Modular program suite for ab initio quantum-chemical and condensed-matter simulations. *The Journal of Chemical Physics* **2020,** *152* (18), 184107.
23. Wang, J.; Wolf, R. M.; Caldwell, J. W.; Kollman, P. A.; Case, D. A., Development and testing of a general amber force field. *Journal of Computational Chemistry* **2004,** *25* (9), 1157-1174.
24. Maier, J. A.; Martinez, C.; Kasavajhala, K.; Wickstrom, L.; Hauser, K. E.; Simmerling, C., ff14SB: Improving the Accuracy of Protein Side Chain and Backbone Parameters from ff99SB. *Journal of Chemical Theory and Computation* **2015,** *11* (8), 3696-3713.
25. Dickson, C. J.; Walker, R. C.; Gould, I. R., Lipid21: Complex Lipid Membrane Simulations with AMBER. *Journal of Chemical Theory and Computation* **2022,** *18* (3), 1726-1736.

26. Jorgensen, W. L.; Chandrasekhar, J.; Madura, J. D.; Impey, R. W.; Klein, M. L., Comparison of simple potential functions for simulating liquid water. *The Journal of Chemical Physics* **1983,** *79* (2), 926-935.
27. Joung, I. S.; Cheatham, T. E., III, Molecular Dynamics Simulations of the Dynamic and Energetic Properties of Alkali and Halide Ions Using Water-Model-Specific Ion Parameters. *The Journal of Physical Chemistry B* **2009,** *113* (40), 13279-13290.
28. Joung, I. S.; Cheatham, T. E., III, Determination of Alkali and Halide Monovalent Ion Parameters for Use in Explicitly Solvated Biomolecular Simulations. *The Journal of Physical Chemistry B* **2008,** *112* (30), 9020-9041.
29. Loncharich, R. J.; Brooks, B. R.; Pastor, R. W., Langevin dynamics of peptides: The frictional dependence of isomerization rates of N-acetylalanyl-N′-methylamide. *Biopolymers* **1992,** *32* (5), 523-535.
30. Berendsen, H. J. C.; Postma, J. P. M.; van Gunsteren, W. F.; DiNola, A.; Haak, J. R., Molecular dynamics with coupling to an external bath. *The Journal of Chemical Physics* **1984,** *81* (8), 3684-3690.
31. Ryckaert, J.-P.; Ciccotti, G.; Berendsen, H. J. C., Numerical integration of the cartesian equations of motion of a system with constraints: molecular dynamics of n-alkanes. *Journal of Computational Physics* **1977,** *23* (3), 327-341.
32. Essmann, U.; Perera, L.; Berkowitz, M. L.; Darden, T.; Lee, H.; Pedersen, L. G., A smooth particle mesh Ewald method. *The Journal of Chemical Physics* **1995,** *103* (19), 8577-8593.
33. Götz, A. W.; Williamson, M. J.; Xu, D.; Poole, D.; Le Grand, S.; Walker, R. C., Routine Microsecond Molecular Dynamics Simulations with AMBER on GPUs. 1. Generalized Born. *Journal of Chemical Theory and Computation* **2012,** *8* (5), 1542-1555.
34. Le Grand, S.; Götz, A. W.; Walker, R. C., SPFP: Speed without compromise—A mixed precision model for GPU accelerated molecular dynamics simulations. *Computer Physics Communications* **2013,** *184*, 374-380.
35. Salomon-Ferrer, R.; Götz, A. W.; Poole, D.; Le Grand, S.; Walker, R. C., Routine Microsecond Molecular Dynamics Simulations with AMBER on GPUs. 2. Explicit Solvent Particle Mesh Ewald. *Journal of Chemical Theory and Computation* **2013,** *9* (9), 3878-3888.
36. Case, D. A.; Cheatham Iii, T. E.; Darden, T.; Gohlke, H.; Luo, R.; Merz Jr, K. M.; Onufriev, A.; Simmerling, C.; Wang, B.; Woods, R. J., The Amber biomolecular simulation programs. *Journal of Computational Chemistry* **2005,** *26* (16), 1668-1688.
37. Ben-Shalom, I. Y.; Lin, Z.; Radak, B. K.; Lin, C.; Sherman, W.; Gilson, M. K., Accounting for the Central Role of Interfacial Water in Protein–Ligand Binding Free Energy Calculations. *Journal of Chemical Theory and Computation* **2020,** *16* (12), 7883-7894.
38. Humphrey, W.; Dalke, A.; Schulten, K., VMD: Visual molecular dynamics. *Journal of Molecular Graphics* **1996,** *14* (1), 33-38.
39. Abramson, J.; Adler, J.; Dunger, J.; Evans, R.; Green, T.; Pritzel, A.; Ronneberger, O.; Willmore, L.; Ballard, A. J.; Bambrick, J., Accurate structure prediction of biomolecular interactions with AlphaFold 3. *Nature* **2024**, 1-3.
